# RNA m^6^A involves in regulation of oxidative stress and apoptosis may via NF-kB pathway in cadmium-induced lung cells

**DOI:** 10.1038/s41420-024-02284-w

**Published:** 2025-01-10

**Authors:** Nan Zhang, Jie Yang, Yuan Zhao, Wenhong Li, Bo Zhao, Rongxian Li, Zuoshun He, Shiyan Gu

**Affiliations:** 1 Institute of Preventive Medicine, School of Public Health, Dali University, No. 22, Wanhua Road, Dali, Yunnan 671000 PR China; 2 College of Engineering, Dali University, Dali, Yunnan 671003 PR China

**Keywords:** Epithelial-mesenchymal transition, Lung cancer

## Abstract

Cadmium has been identified as an environmental pollutant and a carcinogen. N^6^-methyladenosine (m^6^A) plays a crucial role in the development of lung tumors, but the mechanisms remain incompletely clarified. In present study, our data demonstrated that prolonged treatment of 1 μmol/L CdSO_4_ for 40 passages in bronchial epithelial cells (Beas-2B cells) resulted in the development of a malignant phenotype, which manifested as boosted proliferation, migration and invasion capacity as well as apoptosis reduction. Proteomic assay revealed that in passage 40 cells, 350 proteins showed differentially expressed in comparison to control, and these proteins were primarily enriched in Kyoto Encyclopedia of Genes and Genomes (KEGG) pathways of “pathways in cancer” and “Chemical carcinoma-reactive oxygen species”. Moreover, the mRNAs of *Nuclear factor kappa B* (*NF-κB) p65* and *NAD(P)H: quinone oxidoreductase 1 (NQO1)*, the key signaling molecules in these two signaling pathways, were predicted to contain m^6^A modification sites with high confidence. The subsequent experimental results indicated that levels of m^6^A and Fat mass and obesity associated protein (FTO) elevated, while Alkylated DNA repair protein alkB homolog 5 (ALKBH5) and YTH Domain Containing Protein 2 (YTHDC2) reduced with the increasing of cadmium treatment generations. Furthermore, the reduction of m^6^A levels by 3-deazide adenosine (DAA, m^6^A inhibitor) was found to significantly inhibit malignant characteristics of cadmium-induced cells, activate molecules involved in the nuclear factor erythroid 2-related factor 2 (NRF2) signaling pathway, and inhibit the activity of NF-κB. It is also noteworthy that the results based on animals indicate that the relevant indicators and biological changes are partially similar to cell experiments. In detail, m^6^A modification levels in lung tissue were observed to increase while the expressions of FTO, ALKBH5 and YTHDC2 were found to drop. Additionally, immunofluorescence examination illustrated the co-localization of the m^6^A regulatory proteins FTO and YTHDC2 with NF-κB. The presented data collectively suggest that chronic cadmium treatment may impact the m^6^A level through influencing regulatory proteins, which could potentially trigger oxidative stress and apoptosis by regulating transcription factors such as NF-κB and NRF2. In conclusion, our study provides a scientific foundation for understanding cadmium toxicity and offers novel insights for treating cadmium-induced lung diseases.

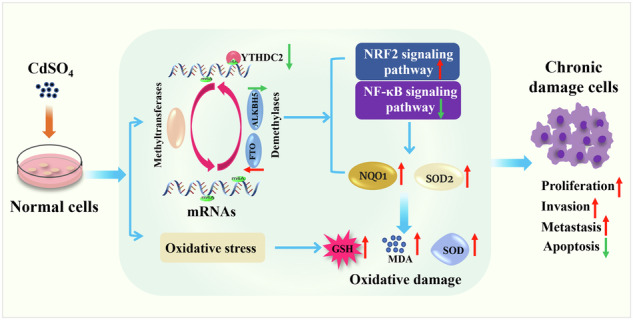

## Introduction

Lung cancer is the most common cause of mortality globally, with around 80,000 new cases diagnosed each year [1]. The main risk factors for lung cancer are environmental exposure and smoking [2]. Cadmium (Cd) is a prevalent heavy metal environmental contaminant and the International Agency for Research on Cancer (IARC) classified cadmium and its compounds as class I carcinogens in 1993 [3]. Humans are mostly exposed to cadmium through ingestion of cadmium-contaminated food and drink, inhaling cadmium-contaminated air and tobacco products [4]. Lung tissue is the primary contact and target organ for cadmium entering through the respiratory tract [5]. Epidemiological investigations have revealed that long-term occupational and environmental cadmium exposure could lead to changes in the metabolism, structure and function of human and animal lung tissues, leading to widespread lung cellular oxidative damage and inflammation, eventually developing into respiratory diseases such as emphysema, chronic obstructive pulmonary disease (COPD), asthma and even lung cancer [6]. In vitro and in vivo studies have shown that short-term high-dose contact with cadmium leads to emphysema, asthma and pulmonary fibrosis, while long-term low-dose cadmium treatment is capable result in cell malignant phenotypes [4, 5].

The extant literature reveals that oxidative damage, cell death and dysregulation of DNA damage repair were all involved in the cadmium toxicity [6–8]. Among of these, oxidative damage is the core mechanism of the occurrence and development of lung diseases, and detailed biological outcomes include cell cycle, uncontrolled proliferation and cell apoptosis [7]. Nuclear Factor κappa-B (NF-κB) and nuclear factor erythroid 2-related factor 2 (NRF2) are key transcription factors involved in apoptosis, cell proliferation and angiogenesis in oxidative stimulation and transcriptional regulation of a series of toxic signals [9]. Under the underlying conditions, NF-κB is retained in the cytoplasm by its inhibitor protein (IκBa). However, upon stimulation, IκBa undergoes rapid phosphorylation by the IkB kinase complex, resulting in the release of NF-κB dimers that translocate to the nucleus and activate the target gene by binding with high affinity to the κB element in the promoter [10, 11]. In the absence of stress, the transcription factor NRF2 is retained in the cytoplasm by interaction with the protein Keap1. Nevertheless, during REDOX stress, NRF2 is released from Keap1 and transferred from the cytoplasm into the nucleus [12, 13]. However, whether NF-κB and NRF2-mediated antioxidant signaling pathways are involved in cadmium-induced chronic lung cell damage remains unclear.

In recent years, the regulatory function of epigenetic modification in the occurrence and development of multiple pathologies has received considerable attention [14]. N^6^-methyladenosine (m^6^A), a prevalent reversible methylation modification on various RNAs, occurs on the adenosine base at the N-6 position, and about 1 ~ 4 m^6^A occurs per thousand adenosine nucleotides in mammalian mRNA [15]. Three types of regulatory proteins, methyltransferases, demethylases and methyl-binding proteins, were involved in production, elimination and functioning of m^6^A modification. In detail, methyltransferases, generally including METTL3, METTL14, WTAP, synergisticly add the m^6^A methylation to the target RNAs. And demethylases, FTO and ALKBH5, remove m^6^A modification, as well methyl-binding proteins, such as YTHDC1/2,YTHDF1/2/3, identify and attach to RNA molecules in an m^6^A-dependent manner [16]. Under the regulation of these three types of proteins, m^6^A modification was involved in the stabilization, transport, cleavage and translation of a variety of RNAs, thus widely affecting cell proliferation and apoptosis, oxidative stress, cell cycle and other pathological and physiological processes [17–19]. Dysregulated m^6^A modification in malignant cells could regulate *NF-κ*B*,NRF2* and *Microtubule-Associated Protein Kinase (MAPK)*, which lead to the abnormality in cell proliferation, survival, migration, and invasion [20]. In cancer cells, loss of METTL3 impairs YTHDF1-mediated Recombinant Sprouty Related, EVH1 Domain Containing Protein 2 (SPRED2) translation, thereby enhancing NF-κB and Signal Transducer and Activator of Transcription 3 (STAT3) activation through the Extracellular Signal-Regulated Kinase (ERK) pathway, leading to increased tumor growth and metastasis [21]. The study demonstrated that the aryl hydrocarbon receptor (AhR) regulates the FTO through transcription, and that FTO then regulates NRF2 in a YTHDF2-m^6^A modification dependent manner, thereby affecting the apoptosis of Bisphenol F (BPF) exposed TM3 cells [22]. However, what are the roles of m^6^A modification and its regulatory proteins in lung cell injury induced by chronic cadmium exposure? Is the mechanism involved related to the NF-κB signaling pathway? These issues have not been clearly defined.

In this study, tumor phenotypes were induced by persistent treatment of low-dose cadmium sulfate (CdSO_4_) in bronchial epithelial cells (Beas-2B). Subsequently, changes of m^6^A modification and its regulatory proteins were detected in these malignant cells. And detailed molecular mechanisms and signaling pathways were determined and analyse with proteomic assay and bioinformatics. Then, based on cadmium treatment mice, changes of m^6^A modification and its regulatory proteins as well as oxidative damage-related molecules were verified in mice lung tissues. Our expected results may elucidate the roles and detailed molecular mechanisms of m^6^A modification in cellular toxicology and tumor phenotype caused by long-term treatment to cadmium. Further, this study also establish a scientific basis for understanding the cadmium carcinogenic mechanisms and offer new insights for prevention and treatment of cadmium-induced lung diseases.

## Results

### Effects of long-term Cadmium treatment on cell morphology and proliferation ability

**Fig. 1:**
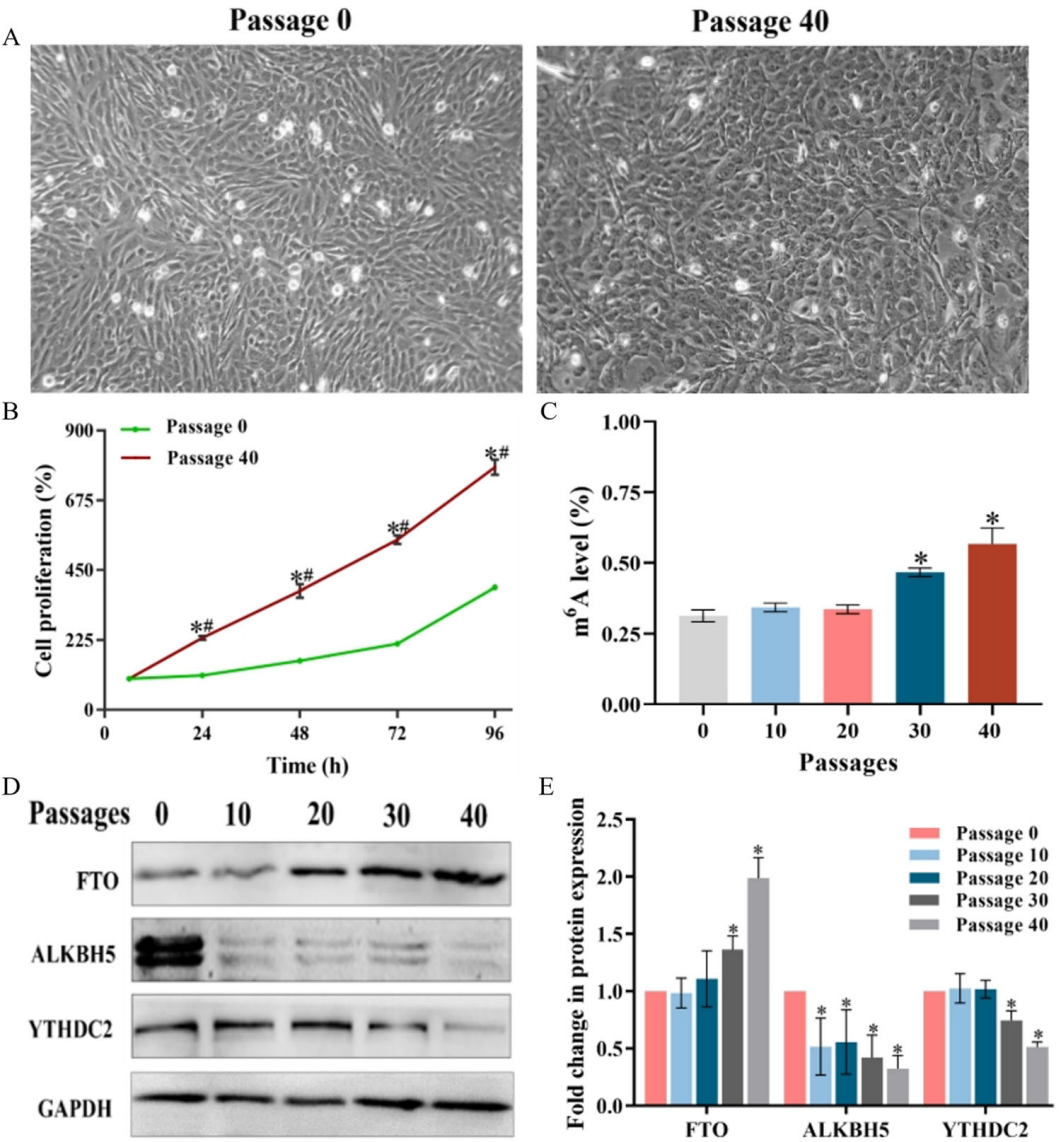
Effect of long-term cadmium treatment on cell morphology, proliferation capacity and m^6^A level. **A** Comparison of cell morphology between Passage 0 and Passage 40 (200×). **B** 72 h growth curves of Passage 0 and Passage 40 cells. **C** The level of m^6^A modification in total RNA of cells in different passages. **D** Immunoblot plot of representative m^6^A regulatory proteins. **E** Quantitative map of protein bands. The symbol “*“ denotes a statistically significant difference (*P* < 0.05) in comparison to the Passage 0 (control group) cells. Three independent experiments were carried out, and data were represented as the means±S.D. “*“ indicates *P* < 0.05 compared with Passage 0 (control group) cells, and the data were obtained by repeating three experiments independently.

As depicted in Fig. 1A, cells in Passage 0 were long spindle-shaped with oval nuclei and cleanly aligned, whereas cells in Passage 40 were disorderly arranged and round in shape, with some nuclei appearing as dense shards and uneven. Figure 1B displayed the proliferation rates of Passage 0 at 24 h, 48 h, 72 h and 96 h as 110.95 ± 2.17%, 158.44 ± 2.42%, 213.24 ± 7.79% and 394.44 ± 4.85%, respectively. The proliferation rates of Passage 40 cells at 24 h, 48 h, 72 h and 96 h were 231.73 ± 6.78%, 382.93 ± 21.41%, 547.45 ± 13.81% and 784.51 ± 19.05%. The proliferation rate at passage 40 was substantially higher than that of the control group at each culture time (*P* < 0.05).

### Effects of long-term Cadmium treatment on m^6^A modification level and its regulatory proteins in cells

Figure 1C illustrated that the m^6^A modification levels of total RNA in cells increased gradually with prolonged exposure to CdSO_4_ at Passage 0, 10, 20, 30 and 40, measuring 0.32 ± 0.02%, 0.35 ± 0.01%, 0.34 ± 0.02%, 0.47 ± 0.01% and 0.57 ± 0.06%, respectively. The m^6^A alteration level in Passage 30 and Passage 40 cells was drastically higher than in Passage 0 (*P* < 0.05). The protein chip screening results indicated a considerable down-regulation of m^6^A demethylase FTO, ALKBH5 and methyl-binding protein YTHDC2 in Passage 30 and Passage 40 compared to the control cells, refer to Appendix 1 for more details. WB results (Fig. 1D, E) indicated that FTO expression considerably increased in Passage 30 and Passage 40, being 1.36 and 1.99 folds of those in Passage 0, respectively (*P* < 0.05). ALKBH5 expression decreased progressively from Passage 10 to Passage 40 compared to Passage 0 (*P* < 0.05) , with values of 0.52, 0.56, 0.42 and 0.32 folds of those in Passage 0, respectively. YTHDC2 protein expression remained consistent in Passage 0, Passage 10 and Passage 20, but declined markedly in Passage 30 and Passage 40 to 0.75 and 0.51 folds of those in Passage 0, respectively, indicating an apparent reduction (*P* < 0.05).

### Effect of m^6^A inhibitor DAA treatment on the malignant phenotype of cells

**Fig. 2:**
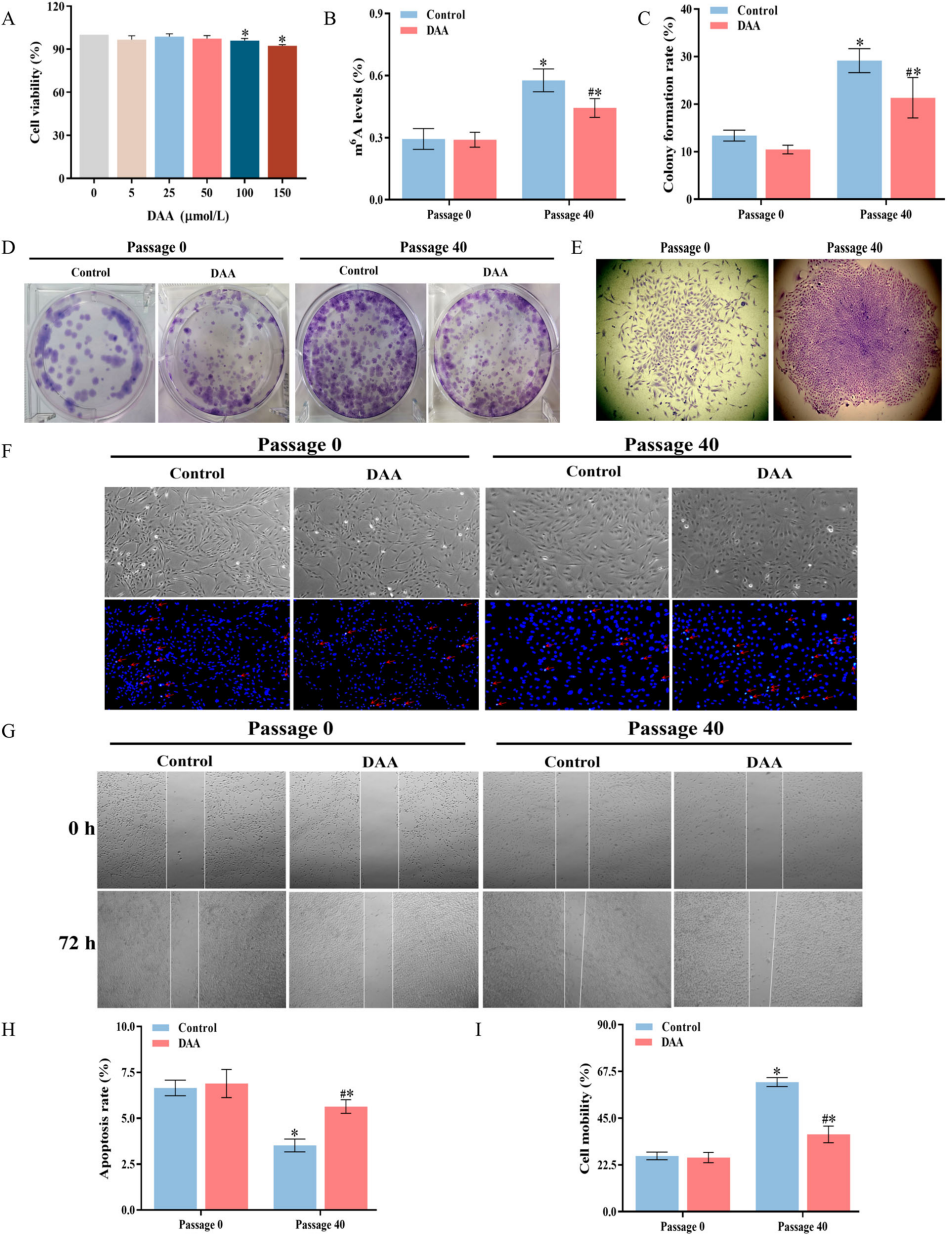
Effect of treatment with the m^6^A inhibitor DAA on the malignant phenotype of cells. Control: No toxic treatment was performed; DAA: 2.5 μmol/L DAA was exposed for 72 h. **A** Effect of DAA on cell viability. **B** Effect of treatment with the m^6^A inhibitor DAA on m^6^A levels on total RNA of cells. **C** Quantitative plot of colony formation rate. **D** Representative image of cell colony formation. **E** Representative images of cell morphology under the microscope of the colony (200×). **F** Representative image of a cell Hoechst staining experiment; red arrows indicate apoptotic cells (200×). **G** Cell scratch healing before and after DAA treatment. **H** Quantitative plot of cell apoptosis. **I** Cell migration rate. *P*-values were calculated by using one-way analysis of variance (ANOVA) and the symbol “*” shows a significant difference (*P* < 0.05) when compared to the “Passage 0-control” group, “#” indicates a remarkable difference (*P* < 0.05) when compared to the “Passage 40-control” group (*P* < 0.05).

Figure 2A demonstrated that cell viability wasn’t particularly impacted by treatment with 0-50 µmol/L DAA (*P* > 0.05). Therefore, we selected the 2.5 μmol/L DAA to treat Passage 0 and Passage 40 cells for 72 h to detect the change of m^6^A level on the total RNA. Figure 2B results indicated a significant rise in the m^6^A level of Passage 40 (0.58 ± 0.03%) compared to the the Passage 0 (0.29 ± 0.03%), but the m^6^A level of Passage 40 decreased greatly after treatment with the inhibitor DAA (0.44 ± 0.03%, *P* < 0.05). The m^6^A level of Passage 0 cells was 0.29 ± 0.03% and DAA treatment did not substantially alter this level (0.29 ± 0.02%, *P* < 0.05). The m^6^A level of Passage 40 cells was 0.58 ± 0.03% and it fell to 0.44 ± 0.03% following DAA treatment. Figure 2C–E displayed the outcomes of the colony formation assay. After Giemsa staining, Passage 0 cell colonies were less in number, light in color and scattered and most of the cells were monolayer adherent growth under microscope. The cell colonies in Passage 40 showed a considerable increase in number and volume compared to the control group and the cells were densely packed and primarily formed stacks when observed under the microscope. Figure 2C exhibited quantitative results indicating that the colony formation rate of Passage 0 cells was 13.40 ± 1.15%, treatment with DAA did not significantly affect the colony formation rate, which was 10.47 ± 0.91% (*P* > 0.05). The colony formation rate of Passage 40 cells was 26.57 ± 4.73%, substantially greater than that of Passage 0 cells (*P* < 0.05). After DAA treatment, the colonies in Passage 40 presented a lighter color, reduced number and a colony formation rate of 21.37 ± 4.24%, substantially lower than that of Passage 40 (*P* < 0.05). Figure 2F revealed that Passage 0 cells showed long spindle-shaped morphology with oval nuclei and consistent fluorescence intensity, some nuclei appeared wrinkled and shiny. In contrast, Passage 40 cells were larger and rounder in shape, the number of apoptotic cells with profound nucleus staining decreased compared to Passage 0. Figure 2H demonstrated that the spontaneous apoptosis rate of Passage 0 was 6.65 ± 0.42%, and treatment with DAA did not significantly impact the apoptosis rate (6.90 ± 0.77%, *P* > 0.05). The apoptosis rate of cells at Passage 40 was 3.52 ± 0.35%, significantly lower than at Passage 0 (*P* < 0.05). Treatment with DAA significantly enhanced the apoptosis rate of cells at Passage 40 to 5.64 ± 0.37% (*P* < 0.05). Figure 2I showed that the cell scratch gradually healed after 72 hours of culture, the migration rate of Passage 0 was 26.84 ± 1.82% and DAA treatment had little effect on it (*P* > 0.05). The wound healing process of Passage 40 cells was more pronounced, with a migration rate of 62.38 ± 2.14%, obviously higher than Passage 0 (*P* < 0.05), the migration rate of Passage 40 was significantly decreased by DAA treatment (37.22 ± 4.01%, *P* < 0.05).

### Proteomics and m^6^A site prediction results

**Fig. 3:**
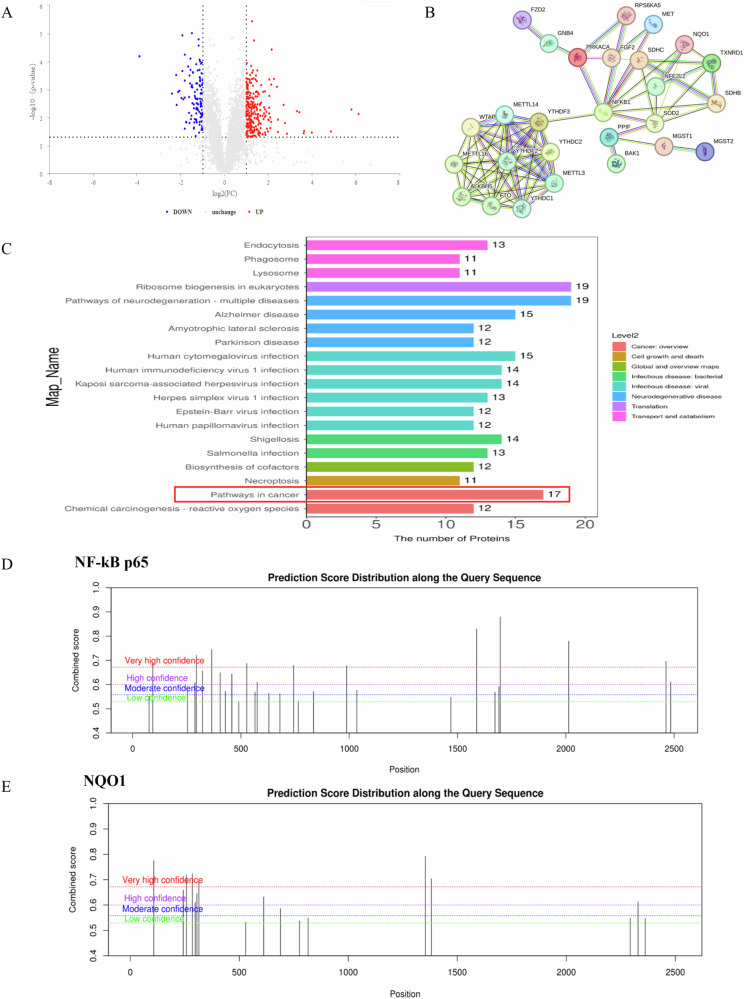
Proteomics and m^6^A site prediction results of Passage 0 and Passage 40 cells. **A** Volcano plot of differentially expressed proteins. **B** Protein interaction network diagram. **C** KEGG signaling pathway map of differentially expressed proteins. **D** Predicted map of m^6^A sites on mRNA of *NF-κB p65*. **E** Predicted map of m6A sites on *NQO1* mRNA.

As shown in TableS S1-S2 (Annex 1), proteomic assay results showed that a total of 4417 common proteins were identified in Passage 0 and Passage 40 cells, and significant changes were detected in 350 of them (|Fold change | ≥2, *P* ≤ 0.05). The volcano plot data (Fig. 3A) illustrated that Passage 40 cells had 236 highly up-regulated proteins and 114 significantly down-regulated proteins, as described in Annex 1. The KEGG signaling pathway map (Fig. 3C) revealed that 13 differentially expressed proteins were enriched in “Pathways in Cancer” and “Chemical carcinoma-reactive oxygen species” which are closely associated with the malignant phenotype of cells or oxidative damage. The protein-protein interaction network (Fig. 3B) revealed that the differentially expressed NF-κB proteins in the signaling pathway directly interacted with various proteins, including NQO1, SOD2 and m^6^A-modified binding protein YTHDF3 (association degree of 0.451 with NF-κB). Furthermore, it was forecasted that there were numerous m^6^A modification sites on the mRNA of *NF-κB p65*,and *NQO1* genes in Fig. 3D, E.

### Effects of m^6^A modification inhibitor DAA on oxidative stress-related indexes of cells

**Fig. 4:**
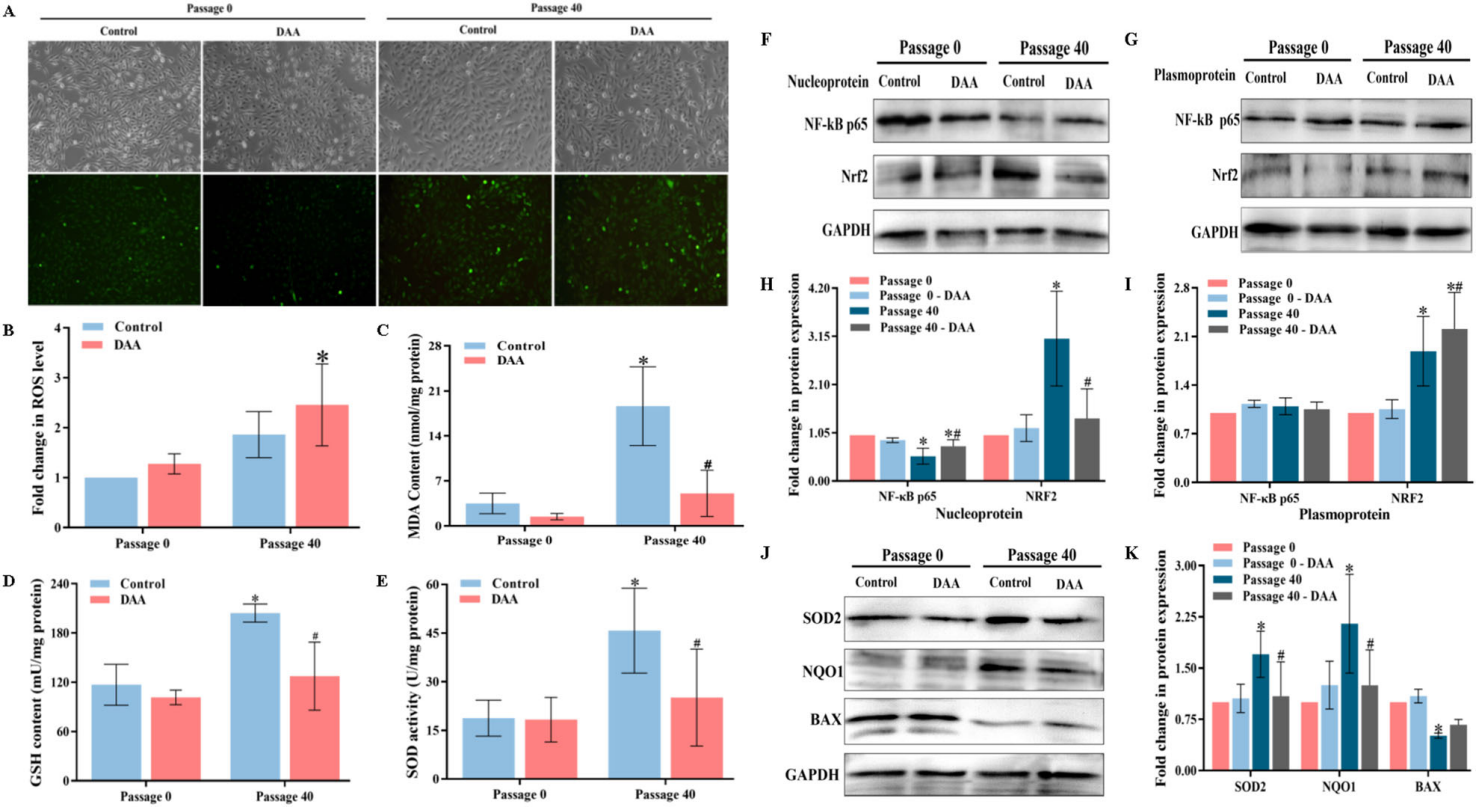
Effects of DAA on cellular oxidative damage. **A** Representative images of ROS in cells before and after DAA treatment (100×). **B** The effect of DAA on ROS in cells. **C** Changes in MDA content in cells after DAA treatment. **D** Effect of DAA on GSH content of cells. **E** Effect of DAA on SOD activity in cells. **F** Immunoblot of representative proteins in the nucleus. **G** Quantitative plot of protein expression in the nucleus. **H** Immunoblot plot of representative proteins in the cytoplasm. **I** Quantitative results of protein expression in the cytoplasm. **J** Immunoblot plots of representative proteins. **K** Quantitative results of protein expression. “*“ reminds markedly difference (*P* < 0.05) when compared to “Passage 0-control” group, “#“ suggests a remarkable difference (*P* < 0.05) when compared with the “Passage 40-control” group (*P* < 0.05).

Figure 4A displayed that Passage 0 had a slight presence of green fluorescence with low intensity, while the fluorescence signal in Passage 40 was stronger. The semi-quantitative results (Fig. 4B) revealed that m^6^A modification inhibitor DAA treatment had no significant effect on the ROS level of Passage 0 cells, which was 1.27-fold of those in Passage 0 (*P* > 0.05). The ROS level of cells in Passage 40 was 1.86 -folds of those in Passage 0, but the difference was not statistically significant (*P* < 0.05), after treatment with DAA, the ROS levels were significantly increased (2.46-fold, *P* < 0.05). MDA concentrations in Passage 0 cells were 3.52 ± 0.93 and 1.46 ± 0.28 nmol/mg protein before and after DAA treatment, respectively. Passage 40 cells had a considerably greater MDA content (18.63 ± 3.53 nmol/mg protein) compared to Passage 0 (*P* < 0.05) while DAA treatment dramatically reduced MDA content (5.08 ± 2.07 nmol/mg protein, *P* < 0.05), as demonstrated in Fig. 4C. There was no significant difference in GSH content in Passage 0 before and after DAA treatment, with values of 117.08 ± 24.91 and 101.65 ± 8.86 mU/mg protein, respectively. The GSH content in Passage 40 was 204.32 ± 11.01 mU/mg protein, significantly greater than in Passage 0 (*P* < 0.05), treatment with DAA dramatically reduced the GSH content in Passage 40 cells to 127.49 ± 41.46 mU/mg protein (*P* < 0.05, Fig. 4D). Figure 4E showed that had no major impact on the SOD activity before and after DAA treatment in Passage 0 which was 18.75 ± 5.55 U/mg protein and 18.31 ± 6.88 U/mg protein, respectively. The SOD activity of Passage 40 was substantially greater than Passage 0 (45.76 ± 13.10 U/mg protein, *P* < 0.05), while the SOD activity obviously decreased to 25.13 ± 14.93 U/mg protein after DAA treatment (*P* < 0.05).

### Effects of m^6^A modification inhibitor DAA on NF-κB and NRF2 signaling pathways

As shown in Fig. 4F–I, the expression level of NF-κB p65 in Passage 40 cells decreased generally compared to Passage 0. In particular, the expression level in the nucleus was 0.54-fold of those in Passage 0 (*P* < 0.05), while the expression in the cytoplasm didn’t differ significantly (1.10-fold, *P* > 0.05). The m^6^A modification inhibitor DAA treatment had little effect on the expression of NF-κB P65 protein in Passage 0, but it notably elevated the expression of NF-κB P65 in nuclear proteins of Passage 40 (0.76-fold, *P* < 0.05). In Passage 40 cells, the NRF2 protein expression was increased, with levels in the nucleus and cytoplasm being 3.10 and 1.89 folds of those in Passage 0, respectively (*P* < 0.05). Treatment with DAA failed to affect the expression of NRF2 in Passage 0, but the nucleus of Passage 40 was drastically reduced by 1.36-fold of those in Passage 0, whereas the protein level in the cytoplasm rose by 2.21-fold, with a statistically difference (*P* < 0.05). Figure 4J, K showed that the SOD2 protein expression in Passage 40 cells was 1.70-fold of those in Passage 0. The inhibitor administration did not impact the protein expression in Passage 0 while the protein level of SOD2 in Passage 40 was markedly decreased to 0.91-fold, with a statistical significance (*P* < 0.05). In Passage 40 cells, the NQO1 protein expression was 2.15-fold of those in Passage 0, treatment with DAA substantially decreased the NQO1 protein expression by 1.25-fold of those in Passage 0 (*P* < 0.05).

### The pathology of lung tissue sections in mice

**Fig. 5:**
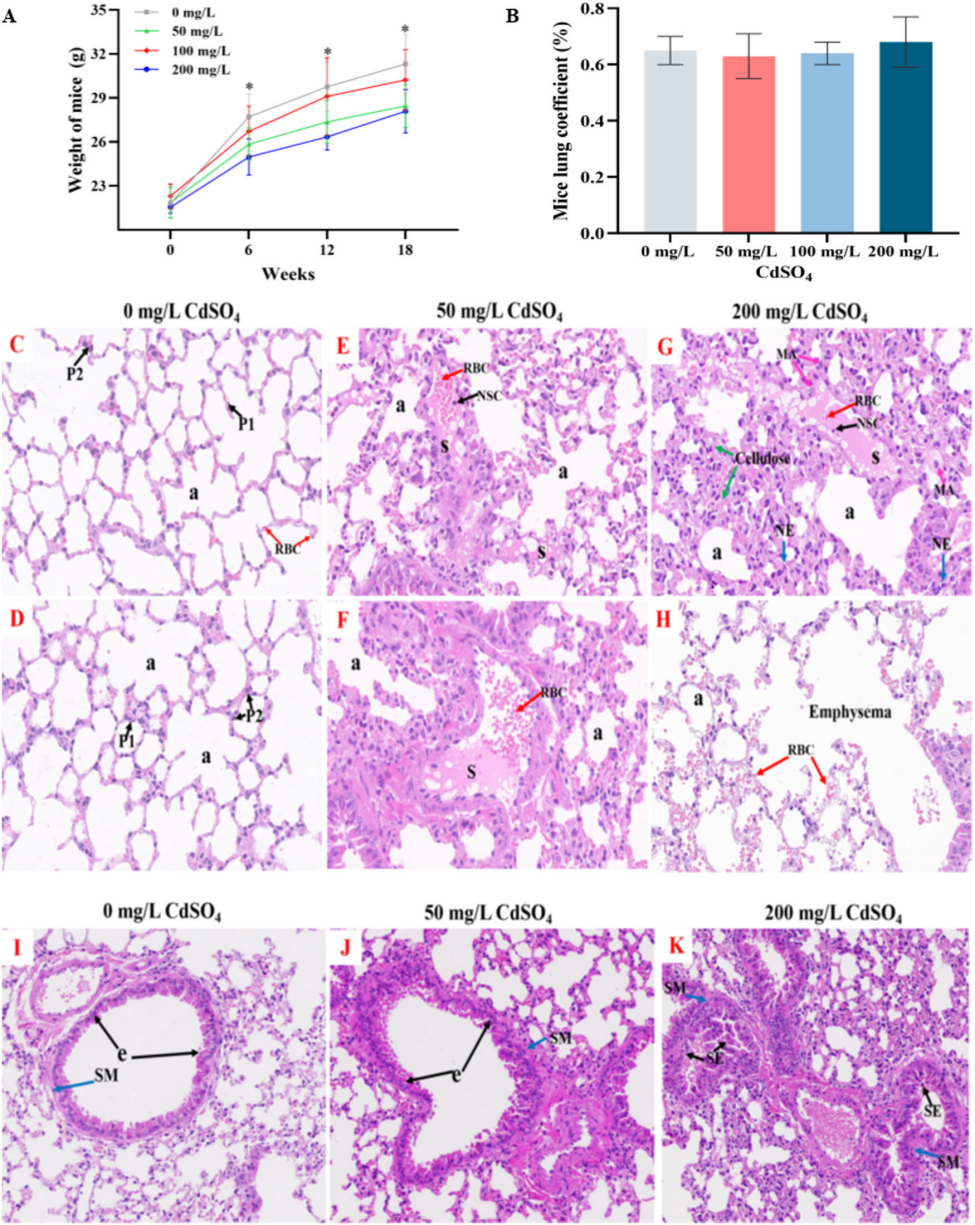
Effects of chronic cadmium treatment on alveolar and bronchial structures. Note: A: alveolar space, P1: flat type I alveolar epithelial cells, P2: cubic type II alveolar epithelial cells, RBC: red blood cells, S: serous fluid, M: macrophages, N: neutrophils, NSC: shed epithelial cells, E: bronchial epithelium, SM: lamina propria smooth muscle, SE: epithelial stratification (200×). **A** The body weight of mice. **B** The lung coefficients of mice. **C**, **D** Control alveolar structure. **E**, **F** Alveolar structure in 50 mg/L cadmium exposed group. **G**, **H** Alveolar structure of 200 mg/L cadmium treatment group. **I** Structure of bronchiole in the control group. **J** Structure of bronchioles in 50 mg/L cadmium treatment group. **K** Bronchiolar structure in 200 mg/L cadmium treatment group.

As shown in Fig. 5A, the body weight of mice increased with the extension of feeding time. In detail, the body weight of mice exposed to 50 and 200 mg/L CdSO_4_ for 6, 12, and 18 weeks was significantly lower than that of the control group (*P* < 0.05). At the termination of the treatment period (18 weeks), the body weight of mice in 0, 50, 100 and 200 mg/L CdSO_4_ treatment group was 31.31 ± 2.01 g, 28.46 ± 1.46 g, 30.23 ± 2.07 g and 28.09 ± 1.48 g, respectively. Furthermore, the lung coefficients for each treatment group was 0.65 ± 0.05%, 0.63 ± 0.08%, 0.64 ± 0.04%, and 0.68 ± 0.09%, respectively, but there was no significant difference among the groups (*P* > 0.05, Fig. 5B). Figure 5C–H illustrates the alveolar structure of the treated group exposed to different doses of cadmium. Thickening of the alveolar gap, enhanced interstitial nuclear processes, dilated and congested blood vessels in the alveolar wall were observed in all treatment groups. The 50 mg/L CdSO_4_ treatment group exhibited an enlarged alveolar wall, dilated and congested blood vessels in the alveolar wall, and serous exudation (S) and red blood cell exudation (red arrow) in some alveolar spaces, predominantly serous exudation, in comparison to the control group. In the group exposed to 200 mg/L of CdSO_4_, blood vessels in the alveolar wall were dilated and congested, local alveoli were visibly dilated, fibrinous were increased presence, and red blood cells and serous exudation in the alveolar space. Besides, a small number of Macrophages (MA) and neutrophils (NE) were present in the alveolar space, along with necrosis and exfoliation of some alveolar epithelial cells (NSC), narrowing and rupture of local alveolar septa, and fusion of adjacent alveoli into large cysts (Emphysema) in 200 mg/L CdSO_4_ treatment group. Figure 5I demonstrated that the standard bronchiole form in the control featured an intact epithelial lining (e) and a consistent layout of smooth muscle (SM) around the bronchiole. Figure 5J–K displays partial detachment of epithelial cells, leading to cell fragmentation and stratification of the bronchial epithelium (SE) and the bronchiole smooth muscle in the 50 mg/L and 200 mg/L CdSO_4_ treatment groups appears to exhibit erratic shifting.

**Fig. 6:**
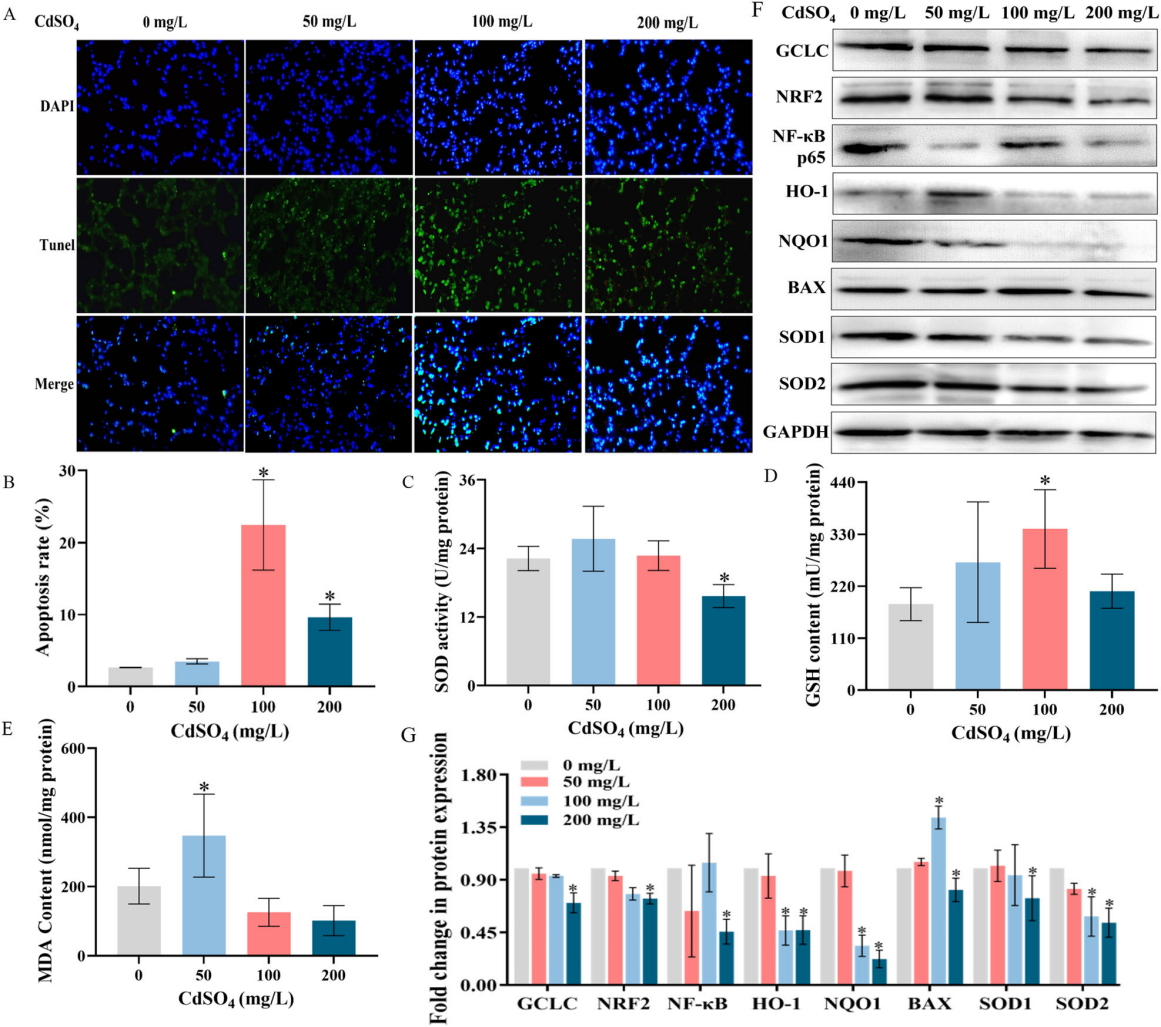
Effects of CdSO_4_ treatment on oxidative damage and apoptosis in lung tissue. **A** Representative image of Tunel staining (200×), blue fluorescence represents the nucleus and green luminous fluorescence represents apoptotic cells. **B** Quantitative plot of apoptosis. **C** Effect of CdSO_4_ treatment on SOD activity in lung tissue. **D** Effect of CdSO_4_ treatment on GSH content in lung tissue. **E** Effect of CdSO_4_ treatment on MDA content in lung tissue. **F** Representative protein immunoblotting of proteins associated with oxidative damage in lung tissue after CdSO_4_ treatment. **G** Quantitative map of protein expression related to oxidative damage in lung tissue. The data were obtained from triplicate independent experiments and are presented as mean ± S.D. *P < 0.05, compared to 0 mg/L CdSO_4_ or control group.

### Effect of CdSO_4_ treatment on apoptosis and oxidative damage in lung tissue

As shown in Fig. 6A, the green fluorescence signal enhanced with the increasing of CdSO_4_ treatment concentrations. In detail, a little green fluorescence was observed in both the control group and 50 mg/L CdSO_4_ group, while strong green fluorescence signals were displayed in 100 mg/L and 200 mg/L CdSO_4_ group with more pronounced nuclear shrinkage and rupture. Fluorescence quantitative analysis in Fig. 6B indicated that the apoptosis rates for the control group, 50 mg/L CdSO_4_ group, 100 mg/L CdSO_4_ group and 200 mg/L CdSO_4_ group were 2.64 ± 0.02%, 3.51 ± 0.37%, 22.45 ± 6.26% and 9.64 ± 1.82%, respectively. The apoptosis rate in the groups exposed to 100 mg/L and 200 mg/L of CdSO_4_ was considerably higher compared to the control group (*P* < 0.05). Figure 6C–E illustrated that MDA significantly increased in the 50 mg/L CdSO_4_ group, SOD activity decreased drastically in the 200 mg/L CdSO_4_ group and GSH increased substantially in the 100 mg/L CdSO_4_ group, indicating a remarkable difference compared with the control (*P* < 0.05). Specifically, SOD activities were 22.26 ± 1.23 U/mg protein, 25.69 ± 3.28 U/mg protein, 22.76 ± 1.50 U/mg protein and 15.68 ± 1.16 U/mg protein in the control group, 50 mg/L, 100 mg/L and 200 mg/L CdSO_4_ treatment group, respectively, as shown in Fig. 6C. The levels of GSH in lung tissues were measured as 182.16 ± 34.89 mU/mg protein, 270.79 ± 67.25 mU/mg protein, 341.17 ± 51.02 mU/mg protein, and 209.65 ± 36.20 mU/mg protein, in the control group and other CdSO_4_ treatment groups (Fig. 6D). Figure 6E displayed the levels of MDA in the control group and those subjected to 50 mg/L, 100 mg/L and 200 mg/L of CdSO_4_, which were 201.15 ± 29.72 nmol/mg protein, 347.15 ± 69.26 nmol/mg protein, 125.64 ± 23.26 nmol/mg protein and 101.53 ± 25.06 nmol/mg protein, respectively. WB results (Fig. 6F, G) showed that the protein expression levels of GCLC, HO-1, NQO1 and SOD2 exhibited a similar trend in response to varying doses of CdSO_4_ treatment. At a concentration of 50 mg/L CdSO_4_ exposure lacked a significant impact on protein expression (*P* < 0.05), while protein expression reduced considerably with increasing CdSO_4_ treatment dose at 100 mg/L and 200 mg/L (*P* < 0.05). Following treatment to 100 mg/L CdSO_4_, the protein expression levels of HO-1, GCLC, NQO1 and SOD2 increased by 0.94, 0.47, 0.34 and 0.59 fold of those in the control, respectively. After dealing with 200 mg/L CdSO_4_, protein expression dropped to 0.70, 0.47, 0.22 and 0.53 fold of those in the control. Similarly, the proteins SOD1 and NRF2 demonstrated comparable patterns when exposed to various levels of CdSO_4_. Protein expression considerably decreased following exposure to 200 mg/L CdSO_4_, with values of 0.71 and 0.74 fold of those in the control (*P* < 0.05), there was no statistically significant difference in protein expression across the other concentrations (*P* < 0.05). The NF-κB p65 protein expression levels in the 50 mg/L, 100 mg/L and 200 mg/L CdSO_4_ treatment groups were 0.63, 1.05 and 0.46 fold of those in the control, respectively. NF-κB p65 expression failed to change significantly after dealing with 50 and 100 mg/L of CdSO_4_ but decreased markedly at a dose of 200 mg/L (*P* < 0.05). The expression of Bax in various concentrations of cadmium was 1.05, 1.43 and 0.81 fold of those in the control, respectively, the differences were statistically significant in 100 and 200 mg/L CdSO_4_ group comparison with the control group (*P* < 0.05).

### Changes in m^6^A modification and regulatory protein levels in lung tissues after CdSO_4_ treatment

**Fig. 7:**
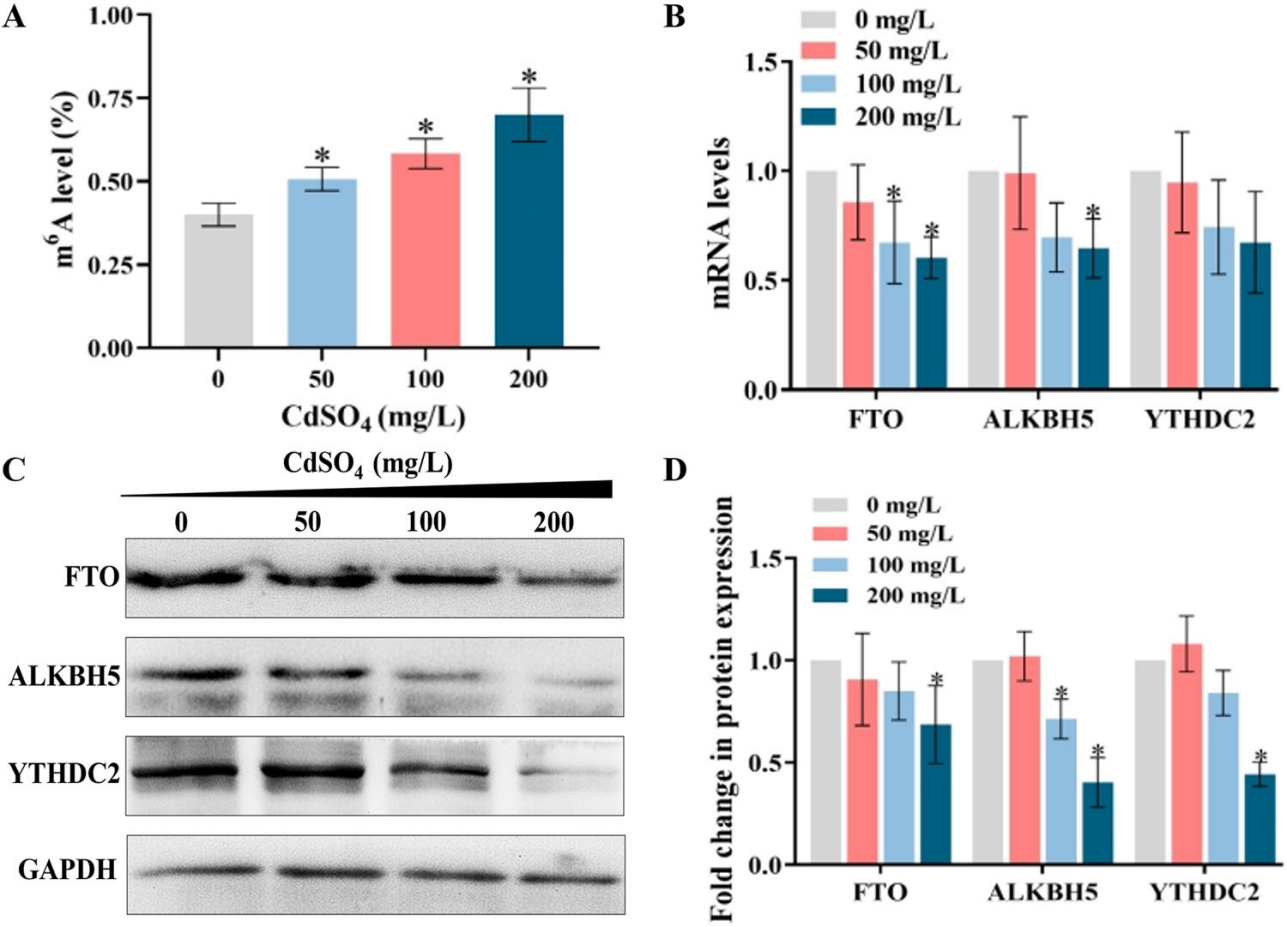
Effect of cadmium treatment on m^6^A modification level. **A** Levels of m^6^A modification on total RNA in lung tissue after dealing with different concentrations of CdSO_4_. **B** mRNA levels of m^6^A regulatory proteins in lung tissues after cadmium treatment. **C** Representative immunoblot of m^6^A regulatory proteins in lung tissue after cadmium treatment. **D** Quantitative map of protein bands. The results were expressed as means ± standard deviations. *P < 0.05, in comparison to the group treated with 0 mg/L CdSO_4_.

Figure 7A displayed that the m^6^A modification levels of total RNA in lung tissue for the control group and 50, 100 and 200 mg/L CdSO_4_ treatment group were 0.40 ± 0.03%, 0.51 ± 0.04%, 0.58 ± 0.04% and 0.70 ± 0.08%, respectively, there was a notable rose in the level of m^6^A alteration as the exposure dose increased (*P* < 0.05). RT-PCR results indicated that the mRNA levels of m^6^A regulatory proteins *Fto*, *Alkbh5* and *Ythdc2* remained unchanged after dealing with 50 mg/L of CdSO_4_, as shown in Fig. 7B. During treatment to 100 mg/L of CdSO_4_, the mRNA levels of *Fto*, *Alkbh5* and *Ythdc2* all decreased to 0.67, 0.70 and 0.74 fold of those in the control, respectively, but only the *Fto* mRNA level differed significantly from the control (*P* < 0.05). Following deal with 200 mg/L CdSO_4_, the mRNA levels of both *Fto, Alkbh5* and *Ythdc2* fall to 0.60, 0.65 and 0.67 fold of those in the control, respectively, the mRNA levels *of Fto and Alkbh5* indicate a remarkable difference compared with the control (*P* < 0.05). The WB results (Fig. 7C, D) were generally in agreement with the PCR. The expression of FTO protein decreased at concentrations of 50, 100 and 200 mg/L of CdSO_4_ treatment to 0.91, 0.85 and 0.69 fold of those in the control, respectively, while only the 200 mg/L CdSO_4_ group revealed a significant difference (*P* < 0.05). Treatment of 50 mg/L CdSO_4_ did not have a notable impact on ALKBH5 protein expression, protein expression descended dramatically to 0.71 and 0.40 fold of those in the control when exposed to 100 and 200 mg/L CdSO_4_, respectively (*P* < 0.05). The YTHDC2 expression in 200 mg/L CdSO_4_ group decreased by 0.44-fold with statistical significance (*P* < 0.05) but had no differences in 50 and 100 mg/L CdSO_4_ group in comparison with the control.

### Immunofluorescence

**Fig. 8:**
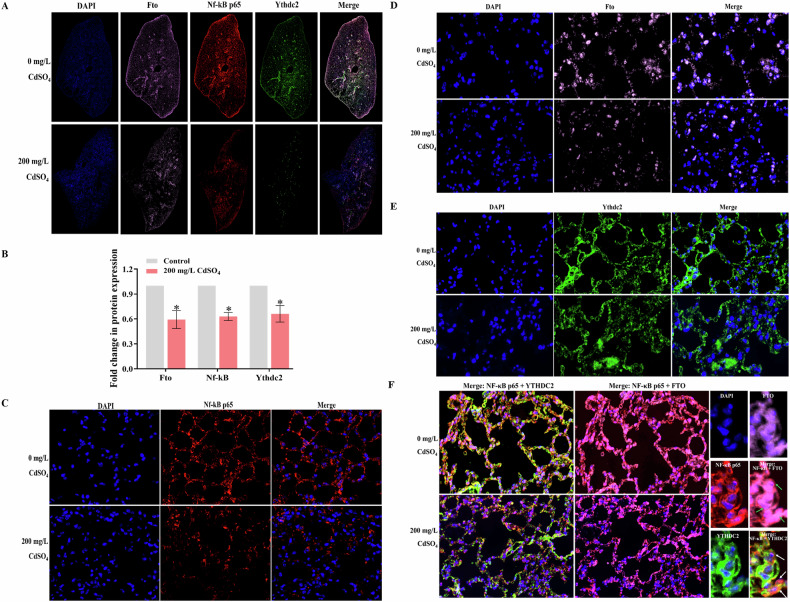
Nuclear and cytoplasmic colocalization of FTO and YTHDC2 with NF-κB. The blue fluorescence represents the nucleus, the pink fluorescence represents the FTO protein, the red fluorescence represents the NF-κB p65 protein, the green fluorescence represents the YTHDC2 protein, and the intensity of fluorescence represents the level of protein expression. The overlaps of others and blue fluorescence represent the localization of the protein in the nucleus, while red and green fluorescence overlap represent the co-localization of YTHDC2 and NF-κB p65 protein in the cytoplasm (white arrow). **A** Immunofluorescence map of different protein expression in lung tissue (10×). **B** Protein immunofluorescence quantitative map. **C** Nuclear and cytoplasmic distribution of Nf-κB p65 protein (400×). **D** Nuclear and cytoplasmic distribution of FTO protein (400×). **E** Nucleoplasmic distribution of YTHDC2 protein (400×). **F** Difference plots of co-localization of FTO and YTHDC2 with NF-κB in different dose groups (300×).

As shown in Fig. 8A, the fluorescence of FTO (pink) and NF-κB p65 (red) in the lung tissues of the control group was widely distributed in the lung tissues with bright colors, while the fluorescence of YTHDC2 protein (green) was mainly concentrated in the bronchioles and was weak in the alveoli. The fluorescence of each protein in the 200 mg/L CdSO_4_ group was significantly reduced, with weaker fluorescence intensity than in the control. The semi-quantitative analysis (Fig. 8B) revealed that deal with to 200 mg/L CdSO_4_ resulted in a noticeable reduction in the average fluorescence intensity of FTO, YTHDC2 and NF-κB p65 proteins by 0.59, 0.63 and 0.66 fold of those in the control, respectively (*P* < 0.05). Figure 8C depicted the distribution of NF-κB p65 protein both in the nucleus and cytoplasm in the control group but mostly located in the cytoplasm with low fluorescence intensity in the 200 mg/L CdSO_4_ group. Figure 8E displayed the distribution of FTO protein in the nucleus and cytoplasm of lung tissues in both the control group and the cadmium-exposed group and the fluorescence intensity was higher in the control group. Figure 8F illustrated the distribution of the YTHDC2 protein in the nucleus and cytoplasm of the control group. Following treatment to 200 mg/L CdSO_4_, YTHDC2 protein was mostly found in the cytoplasm with reduced fluorescence intensity relative to the control. Figure 8F also showed that there was a large amount of overlapping co-localization of Nf-κB p65 (red) and YTHDC2 (green) proteins in the cytoplasm in the control group, while the fluorescence intensity was reduced and protein co-localization was reduced in the 200 mg/L CdSO_4_ group. In addition, the fluorescence of NF-κB p65 (red) and FTO (green) proteins overlapped in the nucleus and cytoplasm in the control group, but there were more co-localized proteins and higher expression levels in the control group.

## Discussion

It is widely accepted that that cadmium and its compounds are common environmental pollutant and carcinogens, in which CdSO_4_ represents the primary form of cadmium is characterized by a lack of visual and olfactory cues, rendering it highly hazardous to human health [23]. Under the prolonged cadmium treatment with a low dose, emerging studies have revealed that some cell lines would appear malignant phenotype, which is characterized by lost contact inhibition, uncontrolled proliferation, reduced apoptosis and strong invasion ability. Also, the emergence of these malignant phenotypes is an important manifestation of chronic cell damage as well as an important link in the occurrence and development of tumors [24–28]. In detail, data from Li et al. [24] have indicated that malignant phenotypes such as proliferation, migration, invasion and anchorage-independent growth in human bronchial epithelial cells after persistent treatment with 2 μmol/L cadmium for 20 weeks. Furthermore, another study identified that bronchial epithelial cells continuously deal with 1 μmol/L cadmium for 16 weeks effectively causing malignant characteristics [25]. In addition to inducing the malignant phenotype of cells, chronic cadmium exposure also induces growth inhibition in animals, oxidative damage, apoptosis, fibrosis and lung coefficient changes in lung tissue [26, 27]. And changes in these events have been considered to be fundamental pathological in the occurrence and development of lung cancer [28]. In addition, it is estimated that the mean cadmium concentrations for the nonsmokers and smokers were 0.4 and 3.0 micrograms/dry wt in healthy human lung tissues [29]_._ At the animal level, the cadmium accumulation concentrations in lung tissue of mice which continuously administrated by 100 and 200 mg/L cadmium chloride for 12 weeks was 0.53 mg/L (4.8 μmol/L) and 1.25 mg/L (11.1 μmol/L), respectively [30]. Based on the above studies, 1 μmol/L CdSO_4_ was selected to treat the bronchial epithelial cells (Beas-2B) and mice were exposed to 50, 100 and 200 mg/L CdSO_4_ by free drinking water in our present study. These concentrations were comparable to the cumulative levels of cadmium in normal lung tissue and consistent with the concentration range of chronic cadmium poisoning reported by WANG [25] and Tai et al. [30]. Our results showed that after 40 generations of continuous treatment with 1 μmol/L CdSO_4_, the cells displayed malignant phenotypes similar to tumor cells, such as morphological changes, uncontrolled proliferation, decreased apoptosis and enhanced invasion ability (detail in Fig. 1-Fig. 2). Our data from mice experiments revealed that treatment with 50,100 mg/L of CdSO_4_ reduced body weight compared with the control group, and 100 and 200 mg/L of CdSO_4_ resulted in emphysema, apoptosis and oxidation-reduction imbalance in the lung tissue (refer to Fig. 5-Fig. 6). Our results together with the previous publication data indicated that cadmium induced chronic lung injury both in vitro cell lines and in vivo mice.

Nevertheless, although the toxicity mechanisms of cadmium have been discussed, their complicated characteristics have presented great obstacles to the prevention and treatment of cadmium toxicity. The latest developments in RNA methylation research provide novel strategy for identifying biomarkers of cadmium-induced lung toxicity. As the most widespread methylation modification in mammal, m^6^A modification has been indicated to involve in the cell and tissue damage induced by cadmium [31, 32]. In detail, results from Qu et al. [31] demonstrated that cadmium-induced islet cell dysfunction, during which m^6^A modification and its regulatory proteins METTL14 and FTO were all significant changes. In addition, Long-term low-dose cadmium chloride treatment in lung epithelial cells could considerably reduce m^6^A modification levels, which may be primarily regulated by the m^6^A demethylase ALKBH5 [33]. In the cadmium treatment rat, the levels of m^6^A and m^6^A regulatory factors (METTL3, METTL14, WTAP and YTHDF2) in renal tissues were dramatically enhanced, and methylated RNA immunoprecipitation sequencing revealed 2615 m^6^A differential expression peaks [32]. In a word, m^6^A modification play crucial roles in the damage of multiple organs and tissues caused by cadmium under the synergistic mediation of multiple regulatory proteins. Our present study found that the level of m^6^A modification and demethylase FTO increased while YTHDC2 and ALKBH5 significantly decreased in Beas-2B cells with the extension of cadmium treatment time (Fig. 1). As the CdSO_4_ treatment dose rose in lung tissue of mouse, m^6^A modification on total RNAs increased, whereas the expressions of FTO, YTHDC2 and ALKBH5 declined (Fig. 7). Further, we also observed that the cadmium-induced malignant phenotype of cells was inhibited by m^6^A inhibitors DAA (Fig. 2). These findings together with the publication data from previous studies, which suggest that regulatory proteins-mediated m^6^A modification might be exploited as independent factors to maintain the malignant phenotype of tumors, indicated that m^6^A modification and its regulatory proteins (FTO, YTHDC2 and ALKBH5) may involve in the process of malignant phenotype induced by cadmium.

The precise mechanism of m^6^A modification and the role of regulatory proteins in cadmium-induced cell and tissue damage remain unclear. A literature review reveals that maintaining oxidative stress homeostasis might assist cancer cells in surviving and counteracting apoptosis, whereas dysfunction of oxidative and antioxidant systems will alter the malignant phenotype of cells [34]. During arsenic-induced cell transformation, m^6^A coordinates the adaptive expression of six key m^6^A-targeted antioxidant enzymes (*SOD1, SOD2, CAT, TXN*, and *GPX1* mRNAs) in a time-dependent manner, promoting the formation and maintenance of abnormal REDOX homeostasis required for arsenic carcinogenesis [35]. In consistent with these notions, our present data revealed an imbalance of the oxidative stress system in cadmium-induced cells and lung tissue (Figs. 4 and 6). However, the specific regulatory mechanisms require further investigation. NF-κB and NRF2 are transcription factors that regulate oxidative stimulation, toxic signals, cell apoptosis, proliferation, angiogenesis and tumor progression. The activation of the NF-κB pathway is linked to cell proliferation and apoptosis resistance [10, 35]. In the initial stages of carcinogenesis, NF-κB may act as a tumor suppressor. Conversely, in a malignant microenvironment, the activation of NF-κB has been demonstrated to confer resistance to apoptosis and stimulate proliferation [10]. Furthermore, studies have demonstrated that alterations in m^6^A modification can influence the invasion and apoptosis of cancer cells by modulating the activation of NF-κB signaling pathways [36–39]. Specifically, cadmium exposure may influence the expression of NRF2 and the activation of nuclear translocation to promote the phosphorylation of NF-κB protein in Sertoli cells [36]. In non-small cell lung cancer, upregulation of METTL3 significantly accelerates the activates of the NF-κB signaling pathway, inducing drug resistance and metastasis of cancer cells [38]. Another study demonstrated that exposure to benzene decreases the m^6^A level in mouse red blood cells and enhances the stability of *NFKBIA* mRNA and controls the NF-κB/CCL3 pathway, ultimately leading to cellular DNA damage, senescence and apoptosis [39]. Our predicted results indicated that there were multiple m^6^A modification sites on the mRNA of *NF-κB p65* (Fig. 3). Subsequent investigations demonstrated that NF-κB was inhibited in cells exposed to chronic cadmium for 40 generations, while NRF2 translocated to the nucleus and promoted the expression of antioxidant proteins NQO1 and SOD2. However, the reduction of m^6^A reversed the expression of these proteins and alleviated oxidative damage and apoptosis. More importantly, our immunofluorescence assay confirmed the colocalization of the m^6^A regulatory proteins FTO and YTHDC2 with NF-κB (Fig. 8). These results together with previous literature reports may suggest that m^6^A participates in regulating cadmium-induced cell malignant phenotype through NF-κB pathways. The specific mechanisms involved in this process may be related to two aspects: one is FTO and YTHDC2 affect the m^6^A modification on *NF-κB* mRNA, thus regulating pathway activation. The other is that FTO and YTHDC2 directly interact with NF-κB protein, which ultimately affect cell proliferation and apoptosis. Nevertheless, the detailed mechanisms of m^6^A modification and its regulatory proteins regulating the NF-κB and NRF2 pathway need to be further explored.

## Conclusion

In conclusion, we identified the roles and mechanisms of m^6^A modification and its regulatory proteins in promoting cadmium-induced malignant phenotype. In this study, a novel model to explain the molecular mechanisms of m^6^A modification have been proposed: cadmium could decrease the level of demethylase ALKBH5, while raising m^6^A modification on RNA, thus influencing transcription factors including NF-κB and NRF2 which regulate oxidative stress and apoptosis, ultimately leading to cell malignant phenotype, which characterized as uncontrolled growth and increased migratory capacity. However, there are still shortcomings in our research, using an inhibitor of m^6^A modification could potentially regulate the m^6^A modification level in a nonspecific manner, and it is essential to target the m^6^A regulatory proteins to selectively manipulate the m^6^A alteration level and elucidate its impact on the malignant phenotype of cells in future investigations. In addition, future studies should clarify the level of m^6^A modification on *NF-κB* and *NRF2* mRNA to understand its regulatory mechanisms on these transcription factors. The direct mechanism of demethylase FTO, ALKBH5 and methyl-binding protein YTHDC2 with NF-κB and NRF2 also needs to be analyzed. Nevertheless, these present results provide new insights into the mechanism of cadmium toxicity and indicating our future research directions.

## Methods

### Cell culture and cadmium treatment

Human bronchial epithelial cells (Beas-2B cells) were purchased from Yuanjing Biotechnology Co., Ltd in 2020. The cell lines were cultured in high glucose Dulbecco’s modified Eagle’s medium (DMEM, Gibco Life Technologies, Grand Island, NY, USA) supplemented with 10% (v/v) fetal bovine serum, 100 mg/mL penicillin and 100 mg/mL streptomycin. The well-grown Beas-2B cells were digested with pancreatic enzyme (Gibco Life Technologies, Grand Island, NY, USA) into a single-cell suspension and inoculated in a 75 cm^2^ culture flask. When the cells adhered to the wall and fused to 40–50%, the culture medium with a final concentration of 1 μmol/L CdSO_4_ was added for 72 h and then passaged, the culture was repeated for 40 passages (about 6 months). At the same time, a control group without CdSO_4_ was set up. At the end of each treatment, the morphology of the cells was observed under a microscope and the cells were routinely frozen every 5 generations for reserve.

### Animal feeding and cadmium treatment

For animal experiments, 8-week-old C57BL/6 J male mice with weighing an average of (20 ± 2) g were used. After one week of adaptive feeding, the mice were randomly assigned to four groups: control group, 50 mg/L CdSO_4_ group, 100 mg/L CdSO_4_ group and 200 mg/L CdSO_4_ group, each containing 7 mice. The mice were provided with an unconstrained regular diet and had unlimited access to food and water during the trial. The control group drank ultra-pure water, whereas the cadmium-exposed groups consumed ultra-pure water with CdSO_4_ for 18 weeks. The animal experiments were approved by Dali University’s Experimental Animal Ethics Committee (approval NO. 2023-P2-204). Animals were housed in cages with a room temperature of 22 ± 2 °C, 50% relative humidity, and a 12-hour light/dark cycle throughout the study. After the last treatment with CdSO_4_, they were starved for 12 hours and then slaughtered the following day. After euthanizing the mice and placing them in a supine position, their chest and abdominal cavities were opened to extract and weigh the entire lung tissue, and the lung coefficient of the mice was calculated (Lung coefficient = lung wet weight (g)/ body weight (kg)×100%). A portion of the lung lobe was promptly preserved in 4% paraformaldehyde for paraffin embedding, the remaining lung lobes were preserved in liquid nitrogen.

### Cell viability was detected by CCK8 assay

CCK8 assay was employed to quantify cell proliferation adopting Yang et al.‘s [33] methods. The cells were inoculated into the standard 96-well culture plate with 1 × 10^3^ cells per well. The culture solution was discarded at 12, 24, 48, 72 and 96 h after inoculation, and then 100 μL culture solution containing CCK8 reagent (Nanjing Jiancheng Technology Co., Ltd., China) with a volume of 10 μL was added to each well. The cells were incubated at 37 °C without light for an additional 3.5 hours, and the absorbance value A_450_ at 450 nm was recorded via an enzyme-linked immunoassay (Multiskan GO, Thermo Fisher Scientific, Inc., Waltham, MA, USA). Cell proliferation rate was calculated and cell growth curve was drawn. Cell proliferation rate (%) = TnA_450_/T12A_450_×100%, TnA_450:_ the absorbance of cells at 450 nm wavelength at different inoculation times, T12A_450_: the absorbance of cells at 450 nm wavelength after 12 h inoculation.

### Cell apoptosis was detected by Hoechst 33258 staining

The well-grown Beas-2B cells were seeded at 2 × 10^4^ cells per well in 6-well plates. After an overnight incubation, the cells were treated with 3-deazidyladenosine (DAA, m^6^A inhibitor, Yuanye Bio-Technology Co., Ltd., Shanghai, China) for 72 hours. Cell apoptosis was subsequently determined following Sun et al.‘s [40] approach, in which 500 uL of fixing solution was delivered to each well for 10 minutes before being thrown away. After washing twice with PBS, 500 uL of Hoechst stain solution was added and incubated for 5 minutes in the dark. Following incubation, the cells were observed under a fluorescence microscope (Olympus Corp, Tokyo, Japan) with an excitation wavelength of 350 nm (ultraviolet light) to recognize apoptosis and take photographs. Rate of cell apoptosis = (number of apoptotic cells / total cells) × 100%.

### Cell proliferation ability was detected by Colony formation assay

Each well was implanted with 1000 cells and incubated for 2 weeks or until most individual colonies contained more than 50 cells. The medium was replaced every 3 days and the cell’s condition was monitored throughout the process. At the end of the culture, the cells were washed 3 times with PBS and fixed in 70% ethanol for 10 min. After that, Giemsa solution (Beyotime Institute of Biotechnology, Jiangsu, China) was added to stain the colonies for 30 min and then thoroughly washed with distilled water. Subsequently, the dried stained colonies were examined and photographed using a light microscope (Olympus Corp, Tokyo, Japan) [33]. The colony count was determined using Image J software (National Institutes of Health, USA), colony formation rate (%) = number of colonies/number of inoculated cells × 100%.

### Cell migration ability was detected by scratch assay

2 × 10^5^ cells per well were inoculated into the 6-well plate. After the cells adhered to the wall, three parallel lines were marked on the cell surface using the tip of a 200 µL pipette gun. Then, the floating cells were gently washed off with PBS, culture medium/cadmium solution was added and the scratched cells were placed under a microscope (Olympus Corp, Tokyo, Japan) to observe whether cell-free scratches appeared, then photos were taken at 0 h and 72 h after scratch [33]. Three distinct sites were chosen to measure the width of the cell-free area and determine the pace of cell migration. Cell migration rate = (0 h scratch width value -72h scratch width value)/0 h scratch width value × 100%.

### The intracellular ROS accumulation was detected by the fluorescence probe

According to the instructions of the ROS detection kit (Beyotime Institute of Biotechnology, Jiangsu, China), the DCFH-DA probe, diluted with a 2000-fold culture medium, was added to each well cell as directed. The cells in each well were incubated in the incubator for 20 minutes. The green fluorescence was observed using a fluorescence microscope (Olympus Corp, Tokyo, Japan) with 488 nm excitation light (blue light) after cleaning the cells twice with PBS. Image J was utilized to measure fluorescence intensity from a randomly selected field of view in a fluorescent microscope [41]. Based on semi-quantitative data, the average fluorescence intensity of ROS = Sum of optical densities / Cell area to be measured.

### Key signaling molecules were identified by proteomic screening and bioinformatics analysis

We utilized Label-free quantitative proteomics technology (Shanghai Zhongke New Life Biotechnology Co., Ltd., China) to analyze the differential changes in Protein expression within the comparison group, following the research methodology outlined by Zhang et al. [42]. The experimental analysis process consists of two primary stages: conducting a mass spectrometry experiment and analyzing the data. The process of mass spectrometry analysis involves protein extraction, peptide enzymatic hydrolysis, liquid chromatography-tandem mass spectrometry (LC-MS/MS), protein identification and quantification, and database retrieval. Following the database search, the data underwent identification number analysis, differential expression analysis, and functional analysis, including KEGG signaling pathway analysis, GO function analysis and protein interaction network PPI analysis. The m^6^A alteration sites were predicted using essential signal molecules as described by Zhao et al. [43]. We obtained the target gene mRNA full gene nucleotide sequence (FASTA genomic sequences) from NCBI. Following that, we pasted the sequence into the SRAMP online server (http://www.cuilab.cn/sramp/) and chose the full transcriptional pattern for predicting m^6^A modification sites.

### The pathological changes of lung tissue were observed by H&E staining

HE staining was performed according to the method described by Wang et al. [44] (Wuhan Saiweier Biotechnology Co., Ltd, China). In brief, paraffin tissue sections were immersed in xylene for 10 minutes and then immersed in gradient ethanol (100%, 90% and 70%) once for 1 min each time. The tissue sections were then placed sequentially in ultrapure water for 1 min, hematoxylin for 15 min and Scott buffer for 8 min. The slides were subsequently rinsed with ultrapure water, and then the tissue sections were sequentially immersed in eosin, gradient ethanol (70%, 90% and 100%) and xylene. After the samples were stained, the slices were sealed and the pathological changes of lung tissue were observed under a microscope. The pathological images were collected and analyzed.

### Apoptosis of lung tissue was detected by Tunel DAB in situ chromography

Tissue apoptosis was identified using the Tunel DAB in situ color development kit (Wuhan Saiweier Biotechnology Co., Ltd, China) [45]. The paraffin sections were placed in an oven at 58 °C for 1.5 h, then immersed in xylene for 30 min, anhydrous ethanol for 5 min, 85% ethanol for 5 min, 75% ethanol for 5 min and distilled water for 5 min. After washing the sections with PBS, 100 μL of Proteinase K working solution (20 μg/mL) was added to each section sample and incubated at 37 °C for 20 min. After incubation, the samples were rinsed three times with PBS. Then, 60 μL of the membrane-breaking solution was dropped to thoroughly penetrate the tissues and incubated at room temperature for 20 min. Afterward, samples were rinsed three times with PBS before adding 50 μL of Equilibration Buffer and incubating for 10 min at room temperature. Subsequently, the buffer was removed and 56 μL of TdT incubation buffer was added to each tissue sample (recombinant TdT enzyme: CF488-dUTP Labeling Mix: Equilibration Buffer=1 µL: 5 µL: 50 µL). After 1 hour of incubation at 37 °C in the dark, the samples were rinsed four times with PBS, and the PBS solution around the samples was wiped with filter paper. The sample was then immersed in a dyeing tank containing DAPI solution and left at room temperature, away from light, for 8 min. The sample was colored and then cleaned three times with PBS to eliminate any surplus liquid. The anti-fluorescence quenching sealing tablets were added to the sealing tablets and the images were immediately observed and recorded using a fluorescence microscope.

### The Nuclear and cytoplasmic colocalization of FTO and YTHDC2 with NF-κB was detected by Immunofluorescence assay

Immunofluorescence detection was carried out in the literature of He et al. [46]. In brief, the sections were successively immersed in the deparaffinization solution I for 10 min, the deparaffinization solution II for 10 min, the deparaffinization solution III for 10 min, absolute ethanol I for 5 min, absolute ethanol II for 5 min, absolute ethanol III for 5 min, distilled water for 5 min for washing and then antigen repair was performed as shown in Table 1. Following the repair, the slides were washed three times in PBS and shaken on a decolorization shaker for 5 min each. The washed sections were immersed in a 3% hydrogen peroxide solution and incubated at room temperature in the dark for 25 minutes. The slides were then cleaned three times with PBS and blocked by dropping BSA for 30 min. After blocking, the first antibody NF-κB p65 was dripped onto the sections, which were then placed flat in a wet box and incubated overnight at 4 °C. The next day, the slides were washed three times with PBS before being incubated for 50 min at room temperature with the matching HRP-labeled secondary antibody. The slides were then washed three times with PBS, dried briefly and TSA (labeled with CY3) was applied drop by drop. The slides were incubated in the dark for 10 min at room temperature. After incubation, the slides were placed in TBST and washed 3 times on a decolorizing shaker for 5 min each time. The tissue pieces were placed in a repair box with antigen repair buffer and heated in a microwave oven over a medium fire for 8 minutes, followed by 7 min on medium and low fire. The second antibody YTHDC2 and the third antibody FTO were incubated in the same order. The slides were stained with DAPI staining solution at room temperature in the dark for 10 min. After washing the slides again, they were sealed with an anti-fluorescence quenching agent, and the images were immediately assessed under a fluorescence microscope.

**Table 1:** Antigen repair condition.

Antigen repair condition	Primary antibody				Secondary antibody	
	Name of antibody	Manufacturer	Catalogue number	Dilution ratio	Name of antibody	Corresponding to the TSA
Microwave medium fire for 8 minutes then stop for 8 minutes before, switching to medium and low fire for 7 minutes	NF-κB p65	Abcam	AB32536	1:5000	HRP labeled goat anti-rabbit IgG	cy3
	YTHDC2	Proteintech	27779-1-AP	1:1000		488
	FTO	Abcam	AB280081	1:500		647

DAPI was excited at a wavelength range of 330–380 nm and emitted light at 420 nm; 488 excitation wavelength 465–495 nm, emission wavelength 515–555 nm; For CY3, the excitation wavelength is 510–560 nm and the emission wavelength is 590 nm; The excitation wavelength of CY5 was 608–648 nm, and the emission wavelength was 672–712 nm.

### mRNA levels were determined by quantitative fluorescence real-time polymerase chain reaction (RT-PCR)

Each treatment group’s total RNA was extracted using a Tiangen Biotech total RNA extraction kit, and DNA was reverse transcribed using a Quant cDNA First Strand Synthesis Kit. RNA and DNA concentrations were determined using an ultra-micro UV-Vis spectrophotometer (Hangzhou Suizhen Biotechnology Co., Ltd.). Samples with ratios of 1.8–2.2 were classified as qualified. Fluorescence quantitative PCR on a QuantStudioTM 3 & 5 (Thermo Fisher Scientific, MA, USA) accomplished after SuperReal fluorescence quantitative premixed kit (Tiangen Biotech, Beijing Co., Ltd., China) reaction system preparation. The qPCR reaction conditions [31] included: pre-denaturation at 95 °C for 15 min, denaturation at 95 °C for 10 S, annealing at 60 °C for 32 S, denaturation and annealing with 35 cycles and the sequences of each primer were listed in Table 2. The mRNAs primer was synthesized by General Bioengineering (Shanghai Generay Biological Engineering Co., Ltd., China).

**Table 2:** Primer sequences and lengths of target genes.

Primer	Primer sequences (5’ → 3’)	Lengths (bp)
Ythdc2	Forward ACCGACTAAGTCAATCTCTTGGT Reverse AGGCTCCTAACAGCATGTTTTG	153
Fto	Forward CTTCACCAAGGAGACTGCTATTTC Reverse CAAGGTTCCTGTTGAGCACTCTG	129
Alkbh5	Forward TCCAGTTCAAGCCTATTCG Reverse CATCTAATCTTGTCTTCCTGAG	171
Gapdh	Forward TCTATAAATTGAGCCCGCAGC Reverse CCAATACGACCAAATCCGTTG	151

Glyceraldehyde-3-phosphate dehydrogenase gene (GAPDH) adjusted target gene and protein mRNA level was calculated using 2^-ΔΔCt^(ΔCt = Ct_mRNA_ - Ct_GAPDH_, ΔΔCt = ΔCt_Treatment_ - ΔCt_Control_).

### Levels of m^6^A modification on total RNA was detected by colorimetric assay

We measured the level of total RNA m^6^A modification using the same approach as Xie et al. [47]. Followed the EpiQuick RNA Methylation Quantification Kit (Epigentek Group Inc., USA) instructions by adding 200 ng of high-quality total RNA, 200 ng of negative control and 2 ng of positive control to strip wells with 80 μL binding solution. These wells were incubated with a sealing film for 90 min (37 °C) before discarding the binding solution and washing three times. Afterward, 50 μL of capture solution was added to each well and left to incubate at room temperature for 60 min. The solution was subsequently removed and the strip wells were cleaned three times. After that, 50 μL of antibody detection solution was added to every well and left to incubate at room temperature for 30 min, then these wells were washed four times and aspirated the washing solution. Then, 50 μL of enhancement solution was applied to the wells and incubated for 30 min at room temperature under seal. We incubated these wells with detection solution for 5 min after 5 washings. A Multiskan GO enzyme calibrator (Thermo Fisher Scientific, Inc., Waltham, MA, USA) measured the optical density OD value at 450 nm after adding the termination solution when it became blue. m^6^A%= [(OD_Sample_-OD_NC_)/S]/[(OD_PC_- OD_NC_)/P]× 100%, where PC: positive control and NC: negative control, S: 200 ng RNA sample, P: 2 ng positive control.

### Protein expressions were detected by western blot

Western blot was used to determine the protein expression levels according to the previous study [48]. The total of 50 μg protein of each sample was successively loaded and separated onto SDS-PAGE gel with 5% and 12% concentrations. Following the electrophoresis process, the proteins present in the gel were transferred onto polyvinylidene fluoride (PVDF) membranes. These membranes were then subjected to a two-hour blocking process using 5% non-fat milk at ambient temperature. The PVDF membranes were then probed overnight at 4 °C with primary antibodies (Bioss Biotechnology Co., Ltd. Beijing, China) against the target protein and GAPDH, details in Table 3. The following day, membranes were washed three times with washing buffer and incubated with a secondary antibody (1:6000, Zhongshan Jinqiao Biotechnology Co., Ltd., Beijing, China) at room temperature for 1 hour. The immuno-reactivity zone was acquired by enhanced chemiluminescence reagent (Millipore, Temecula, CA) and image acquisition was conducted with JS-M6 chemiluminescence imaging system. Relative protein expression = gray value of the target bands/gray value of the GAPDH, following by correcting with the protein expression in the control group. Fold change of protein expression was used to express protein levels.

**Table 3:** Target protein manufacturers and dilutions.

Name of primary antibody	Manufacturer	Catalogue number	Dilution ratio	Host	weight (kDa)
YTHDC2 Polyclonal antibody	Proteintech Group, Inc	27779-1-AP	1:2000	Rabbit	160
FTO Polyclonal antibody		27226-1-AP	1:2000		58
ALKBH5 Polyclonal antibody		16837-1-AP	1:2000		40-50
Bax Polyclonal antibody		50599-2-Ig	1:2000		21
NRF2 Recombinant antibody		16396-1-AP	1:1000		110
SOD2 Polyclonal antibody		24127-1-AP	1:5000		25
NQO1 Polyclonal antibody		11451-1-AP	1:2000		31
GAPDH Polyclonal antibody		10494-1-AP	1:5000		36
Recombinant-NF-κB p65 monoclonal antibody	Abcam	AB32536	1:2000		65

### Oxidative stress related indicators were detected by colorimetric assay

After designed treatments, total protein of cells and tissues were collected and their protein concentrations were detected with BCA kits (Beyotime Biotechnology, Jiangsu, China). Sample loading and incubation were carried out according to the instructions of the total SOD activity detection kit, GSH-PX detection kit and MDA detection kit (Beyotime Biotechnology, Jiangsu, China) and the method of Qu. et al. [31]. The optical density was measured at 450, 340 and 532 nm wavelengths by a multifunctional fluorescent microplate reader, and the activities of SOD and GSH-PX and the content of MDA were calculated according to the instructions.

### Statistical analysis

Each experiment was carried out with at least three independent replicates, and the data were presented as mean ± standard deviation (SD). Statistical differences were analyzed using the Statistical Program for the Social Sciences (SPSS 17). The statistical analysis utilized one-way analysis of variance (ANOVA) and the Least Significant Difference (LSD) *t*-test to compare several groups. The nonparametric Kruskal-Wallis test was applied to the raw data when variance heterogeneity existed. The significance level was established at 0.05 and all tests were two-sided.

## Supplementary information

Annex 1

Annex 2

## Data Availability

Data will be made available on request.
