# Supplementary material for: RNA m6A involves in regulation of oxidative stress and apoptosis may via NF-kB pathway in cadmium-induced lung cells
Source: Cell Death Discov. 2025 Jan 10;11:4. doi: 10.1038/s41420-024-02284-w (PMC11723944; doi:10.1038/s41420-024-02284-w)
Supplement: Supplementary file 1 — Annex 1 [file 41420_2024_2284_MOESM1_ESM.docx]

**Table S1. Differentially expressed protein (Upregulated)**

| Protein Name | Passage40/  Passage0 | t test p value | Protein Name | Passage40/  Passage0 | t test p value |
| --- | --- | --- | --- | --- | --- |
| Myelin expression factor 2 （MYEF2） | 70.743 | 0.008 | V-type proton ATPase 116 kDa subunit a 1（ATP6V0A1） | 2.510 | 0.000 |
| Neural cell adhesion molecule L1（L1CAM） | 55.554 | 0.005 | CD63 antigen（CD63） | 3.146 | 0.024 |
| Mitochondrial import inner membrane translocase subunit Tim9（TIMM9） | 2.428 | 0.003 | Nicotinamide phosphoribosyltransferase（NAMPT） | 2.481 | 0.014 |
| Complement decay-accelerating factor（CD55） | 15.829 | 0.035 | Flotillin-1（FLOT1） | 2.481 | 0.008 |
| Superoxide dismutase [Mn], mitochondrial（SOD2） | 12.441 | 0.030 | NAD kinase 2, mitochondrial（NADK2） | 2.480 | 0.036 |
| Aminopeptidase N（ANPEP） | 12.242 | 0.036 | Protein FAM136A（FAM136A） | 2.471 | 0.000 |
| Synaptogyrin-2（SYNGR2） | 10.825 | 0.006 | RNA-binding protein 28（RBM28） | 2.469 | 0.001 |
| E3 ubiquitin-protein ligase NEDD4-like（NEDD4L） | 9.931 | 0.006 | Long-chain-fatty-acid--CoA ligase 1（ACSL1） | 2.468 | 0.015 |
| Insulin-like growth factor-binding protein 7（IGFBP7） | 7.249 | 0.038 | GPI transamidase component PIG-S（PIGS） | 2.502 | 0.004 |
| CD82 antigen（CD82） | 6.827 | 0.016 | 5-nucleotidase（NT5E） | 4.290 | 0.024 |
| Nicotinamide/nicotinic acid mononucleotide adenylyltransferase 1（NMNAT1） | 2.445 | 0.036 | NADPH:adrenodoxin oxidoreductase, mitochondrial（FDXR） | 6.517 | 0.004 |
| Solute carrier family 2, facilitated glucose transporter member 6（SLC2A6） | 5.479 | 0.033 | 2,4-dienoyl-CoA reductase [(3E)-enoyl-CoA-producing], mitochondrial（DECR1） | 2.436 | 0.003 |
| WD repeat-containing protein 46（WDR46） | 2.406 | 0.003 | Antigen peptide transporter 2（TAP2） | 4.264 | 0.010 |
| Syntenin-1（SDCBP） | 5.424 | 0.012 | Argininosuccinate synthase（ASS1） | 29.173 | 0.033 |
| Mitochondrial import inner membrane translocase subunit Tim22（TIMM22） | 2.466 | 0.007 | 4F2 cell-surface antigen heavy chain（SLC3A2） | 2.448 | 0.031 |
| Aldehyde dehydrogenase, mitochondrial（ALDH2） | 5.256 | 0.001 | Dehydrogenase/reductase SDR family member 7（DHRS7） | 2.424 | 0.002 |
| NADP-dependent malic enzyme（ME1） | 4.850 | 0.000 | Antigen peptide transporter 1（TAP1） | 4.078 | 0.013 |
| Coiled-coil domain-containing protein 137（CCDC137） | 4.481 | 0.000 | Poly(A) RNA polymerase, mitochondrial（MTPAP） | 2.423 | 0.000 |
| Probable global transcription activator SNF2L2（SMARCA2） | 4.404 | 0.001 | Serine beta-lactamase-like protein LACTB, mitochondrial（LACTB） | 2.422 | 0.000 |
| CDGSH iron-sulfur domain-containing protein 1（CISD1） | 4.403 | 0.015 | Transferrin receptor protein 1（TFRC） | 2.417 | 0.022 |
| Outer mitochondrial transmembrane helix translocase（ATAD1） | 2.396 | 0.000 | L-2-hydroxyglutarate dehydrogenase, mitochondrial（L2HGDH） | 2.405 | 0.015 |
| Melanotransferrin（MELTF） | 4.177 | 0.007 | Stomatin（STOM） | 4.145 | 0.000 |
| Aspartyl/asparaginyl beta-hydroxylase（ASPH） | 4.146 | 0.002 | Synaptophysin-like protein 1（SYPL1） | 2.390 | 0.002 |
| Membrane-associated tyrosine- and threonine-specific cdc2-inhibitory kinase（PKMYT1） | 2.387 | 0.028 | Phosphatidylinositol 4-kinase type 2-alpha（PI4K2A） | 2.389 | 0.004 |
| Uridine phosphorylase 1（UPP1） | 4.125 | 0.005 | Unconventional myosin-Ib（MYO1B） | 2.388 | 0.004 |
| Leucyl-cystinyl aminopeptidase（LNPEP） | 2.378 | 0.005 | Large neutral amino acids transporter small subunit 1（SLC7A5） | 2.424 | 0.047 |
| U3 small nucleolar RNA-associated protein 6 homolog（UTP6） | 4.031 | 0.012 | Solute carrier family 52, riboflavin transporter, member 2（SLC52A2） | 2.384 | 0.035 |
| Cell surface hyaluronidase（CEMIP2） | 3.967 | 0.032 | DNA ligase 3（LIG3） | 3.658 | 0.043 |
| Transcription factor AP-2-alpha（TFAP2A） | 3.870 | 0.025 | Translocating chain-associated membrane protein 1（TRAM1） | 2.366 | 0.012 |
| NAD(P)H dehydrogenase [quinone] 1（NQO1） | 3.808 | 0.006 | [F-actin]-monooxygenase MICAL2（MICAL2） | 2.353 | 0.007 |
| Tumor necrosis factor alpha-induced protein 2（TNFAIP2） | 3.676 | 0.009 | Transmembrane emp24 domain-containing protein 3（TMED3） | 2.350 | 0.049 |
| Ferroptosis suppressor protein 1（AIFM2） | 2.337 | 0.003 | High affinity copper uptake protein 1（SLC31A1） | 2.338 | 0.020 |
| Nucleolar protein 10（NOL10） | 3.625 | 0.035 | Ferritin heavy chain（FTH1） | 2.330 | 0.027 |
| 2-oxoadipate dehydrogenase complex component E1（DHTKD1） | 3.620 | 0.010 | V-type proton ATPase 116 kDa subunit a 1（ATP6V0A1） | 2.510 | 0.000 |
| Probable ATP-dependent RNA helicase DDX52（DDX52） | 3.610 | 0.001 | GPI transamidase component PIG-S（PIGS） | 2.502 | 0.004 |
| 2-iminobutanoate/2-iminopropanoate deaminase（RIDA） | 3.608 | 0.005 | WD repeat-containing protein 43（WDR43） | 2.327 | 0.020 |
| Intercellular adhesion molecule 1（ICAM1） | 3.523 | 0.004 | RRP12-like proteinR（RP12） | 2.327 | 0.000 |
| A-kinase anchor protein 1, mitochondrial（AKAP1） | 3.513 | 0.007 | Tapasin（TAPBP） | 3.352 | 0.009 |
| Hepatocyte growth factor receptor （MET） | 3.470 | 0.011 | Protein FAM136A（FAM136A） | 2.471 | 0.000 |
| Anion exchange protein 2（SLC4A2） | 3.405 | 0.001 | Flotillin-1（FLOT1） | 2.481 | 0.008 |
| Nicotinamide phosphoribosyltransferase（NAMPT） | 2.481 | 0.014 | RNA-binding protein 28（RBM28） | 2.469 | 0.001 |
| Urokinase plasminogen activator surface receptor（PLAUR） | 3.350 | 0.040 | NAD kinase 2, mitochondrial（NADK2） | 2.480 | 0.036 |
| Lysosome-associated membrane glycoprotein 2（LAMP2） | 3.211 | 0.003 | Long-chain-fatty-acid--CoA ligase 1（ACSL1） | 2.468 | 0.015 |
| Nicotinamide/nicotinic acid mononucleotide adenylyltransferase 1（NMNAT1） | 2.445 | 0.036 | Mitochondrial import inner membrane translocase subunit Tim22（TIMM22） | 2.466 | 0.007 |
| Sulfide:quinone oxidoreductase, mitochondrial（SQOR） | 3.141 | 0.017 | 4F2 cell-surface antigen heavy chain（SLC3A2） | 2.448 | 0.031 |
| Ribosomal oxygenase 1（RIOX1） | 3.125 | 0.009 | Polyhomeotic-like protein 2（PHC2） | 3.005 | 0.035 |
| Bifunctional methylenetetrahydrofolate dehydrogenase/cyclohydrolase, mitochondrial（MTHFD2） | 3.121 | 0.005 | 2,4-dienoyl-CoA reductase [(3E)-enoyl-CoA-producing], mitochondrial（DECR1） | 2.436 | 0.003 |
| U3 small nucleolar RNA-associated protein 4 homolog（UTP4） | 3.107 | 0.003 | Mitochondrial import inner membrane translocase subunit Tim9（TIMM9） | 2.428 | 0.003 |
| Inositol 1,4,5-trisphosphate receptor type 3（ITPR3） | 3.102 | 0.037 | Trophoblast glycoprotein（TPBG） | 2.909 | 0.040 |
| ATP-dependent RNA helicase DDX24（DDX24） | 3.083 | 0.004 | Large neutral amino acids transporter small subunit 1（SLC7A5） | 2.424 | 0.047 |
| Phosphoenolpyruvate carboxykinase [GTP], mitochondrial（PCK2） | 3.034 | 0.016 | Poly(A) RNA polymerase, mitochondrial（MTPAP） | 2.423 | 0.000 |
| Dehydrogenase/reductase SDR family member 7（DHRS7） | 2.424 | 0.002 | Serine beta-lactamase-like protein LACTB, mitochondrial（LACTB） | 2.422 | 0.000 |
| Plasminogen activator inhibitor 2（SERPINB2） | 2.999 | 0.045 | Transferrin receptor protein 1（TFRC） | 2.417 | 0.022 |
| HLA class I histocompatibility antigen, A alpha chain（HLA-A） | 2.994 | 0.001 | WD repeat-containing protein 46（WDR46） | 2.406 | 0.003 |
| Interferon-induced transmembrane protein 3（IFITM3） | 2.956 | 0.014 | Outer mitochondrial transmembrane helix translocase（ATAD1） | 2.396 | 0.000 |
| Ferritin light chain（FTL） | 2.931 | 0.018 | Synaptophysin-like protein 1（SYPL1） | 2.390 | 0.002 |
| 3-ketoacyl-CoA thiolase, mitochondrial（ACAA2） | 2.929 | 0.007 | Phosphatidylinositol 4-kinase type 2-alpha（PI4K2A） | 2.389 | 0.004 |
| Sequestosome-1（SQSTM1） | 2.923 | 0.035 | Unconventional myosin-Ib（MYO1B） | 2.388 | 0.004 |
| Membrane-associated tyrosine- and threonine-specific cdc2-inhibitory kinase（PKMYT1） | 2.387 | 0.028 | L-2-hydroxyglutarate dehydrogenase, mitochondrial（L2HGDH） | 2.405 | 0.015 |
| Galectin-3-binding protein（LGALS3BP） | 2.898 | 0.010 | Solute carrier family 52, riboflavin transporter, member 2（SLC52A2） | 2.384 | 0.035 |
| Phosphoserine aminotransferase（PSAT1） | 2.871 | 0.012 | CD63 antigen（CD63） | 3.146 | 0.024 |
| Adhesion G protein-coupled receptor E5（ADGRE5） | 2.864 | 0.001 | Translocating chain-associated membrane protein 1（TRAM1） | 2.366 | 0.012 |
| Carnitine O-palmitoyltransferase 1, liver isoform（CPT1A） | 2.857 | 0.002 | [F-actin]-monooxygenase MICAL2（MICAL2） | 2.353 | 0.007 |
| Microsomal glutathione S-transferase 1（MGST1） | 2.848 | 0.008 | Protein HEG homolog 1（HEG1） | 2.829 | 0.027 |
| Leucyl-cystinyl aminopeptidase（LNPEP） | 2.378 | 0.005 |  |  |  |
| Transmembrane emp24 domain-containing protein 3（TMED3） | 2.350 | 0.049 | High affinity copper uptake protein 1（SLC31A1） | 2.338 | 0.020 |
| Microsomal glutathione S-transferase 2（MGST2） | 2.826 | 0.003 | Ferroptosis suppressor protein 1（AIFM2） | 2.337 | 0.003 |
| CD70 antigen（CD70） | 2.823 | 0.000 | Protein AATF（AATF） | 3.189 | 0.001 |
| Methylcrotonoyl-CoA carboxylase subunit alpha, mitochondrial（MCCC1） | 2.812 | 0.006 | WD repeat-containing protein 43（WDR43） | 2.327 | 0.020 |
| Type 1 phosphatidylinositol 4,5-bisphosphate 4-phosphatase（PIP4P1） | 2.801 | 0.011 | SH3 domain-containing kinase-binding protein 1（SH3KBP1） | 2.031 | 0.000 |
| HLA class I histocompatibility antigen, B alpha chain（HLA-B） | 2.801 | 0.001 | Glycerol-3-phosphate dehydrogenase, mitochondrial（GPD2） | 2.020 | 0.004 |
| Ribosome biogenesis regulatory protein homolog（RRS1） | 2.797 | 0.001 | Neutral cholesterol ester hydrolase 1（NCEH1） | 2.025 | 0.005 |
| DNA topoisomerase 2-alpha（TOP2A） | 2.789 | 0.026 | Contactin-associated protein 1（CNTNAP1） | 2.978 | 0.031 |
| Neutral amino acid transporter B(0)（SLC1A5） | 2.778 | 0.005 | Mediator of DNA damage checkpoint protein 1（MDC1） | 2.017 | 0.004 |
| Lipid droplet-associated hydrolase（LDAH） | 2.772 | 0.011 | Endoribonuclease LACTB2（LACTB2） | 2.017 | 0.000 |
| Thioredoxin reductase 1, cytoplasmic（TXNRD1） | 2.760 | 0.028 | Glycerol-3-phosphate dehydrogenase, mitochondrial（GPD2） | 2.020 | 0.004 |
| NPC intracellular cholesterol transporter 1（NPC1） | 2.732 | 0.004 | 60S ribosomal protein L7-like 1（RPL7L1） | 2.001 | 0.008 |
| Multidrug resistance-associated protein 1（ABCC1） | 2.712 | 0.001 | Suppressor of SWI4 1 homolog（PPAN） | 2.013 | 0.009 |
| Peptidyl-prolyl cis-trans isomerase F, mitochondrial（PPIF） | 2.708 | 0.000 | Ribosome biogenesis protein NSA2 homolog（NSA2） | 2.043 | 0.001 |
| Cytochrome b5（CYB5A） | 2.707 | 0.021 | Ferritin heavy chain（FTH1） | 2.330 | 0.027 |
| Sphingosine-1-phosphate lyase 1（SGPL1） | 2.682 | 0.000 | RRP12-like proteinR（RP12） | 2.327 | 0.000 |
| Transmembrane protein 50A（TMEM50A） | 2.046 | 0.017 | Ras-related protein Rab-7a（RAB7A） | 2.013 | 0.001 |
| Core histone macro-H2A.2（MACROH2A2） | 2.674 | 0.047 | Bcl-2 homologous antagonist/killer（BAK1） | 2.038 | 0.009 |
| Medium-chain specific acyl-CoA dehydrogenase, mitochondrial（ACADM） | 2.671 | 0.002 | Probable ATP-dependent RNA helicase DDX27（DDX27） | 2.032 | 0.002 |
| Glycogen phosphorylase, liver form（PYGL） | 2.638 | 0.003 | Frizzled-2（FZD2） | 2.004 | 0.001 |
| Ribosome biogenesis protein WDR12（WDR12） | 2.052 | 0.001 | Beta-2-microglobulin（B2M） | 2.676 | 0.008 |
| Choline transporter-like protein 1（SLC44A1） | 2.552 | 0.032 | Ras-related protein Rab-8B（RAB8B） | 2.050 | 0.000 |
| Sigma intracellular receptor 2（TMEM97） | 2.606 | 0.028 | Aprataxin（APTX） | 2.049 | 0.001 |
| Electrogenic aspartate/glutamate antiporter SLC25A12, mitochondrial（SLC25A12） | 2.596 | 0.006 | Tryptophan--tRNA ligase, mitochondrial（WARS2） | 2.038 | 0.001 |
| Pyrroline-5-carboxylate reductase 1, mitochondrial（PYCR1） | 2.573 | 0.004 | U3 small nucleolar RNA-associated protein 14 homolog A（UTP14A） | 2.034 | 0.004 |
| Translocation protein SEC63 homolog（SEC63） | 2.570 | 0.005 | Flotillin-2（FLOT2） | 2.607 | 0.010 |
| Protein canopy homolog 3（CNPY3） | 2.559 | 0.004 | Cohesin subunit SA-1（STAG1） | 2.555 | 0.024 |
| Palmitoyl-protein thioesterase ABHD10, mitochondrial（ABHD10） | 2.064 | 0.001 | CDGSH iron-sulfur domain-containing protein 3, mitochondrial（CISD3） | 2.059 | 0.038 |
| Isocitrate dehydrogenase [NAD] subunit gamma, mitochondrial（IDH3G） | 2.046 | 0.003 | Three-prime repair exonuclease 1（TREX1） | 2.053 | 0.050 |
| Peroxiredoxin-4（PRDX4） | 2.550 | 0.000 | Protein LYRIC（MTDH） | 2.037 | 0.033 |
| Periodic tryptophan protein 2 homolog（PWP2） | 2.545 | 0.045 | Thiosulfate sulfurtransferase（TST） | 2.530 | 0.002 |
| Stromal cell-derived factor 2（SDF2） | 2.532 | 0.016 | Endoglin（ENG） | 2.068 | 0.030 |
| 28S rRNA (cytosine-C(5))-methyltransferase（NSUN5） | 2.066 | 0.002 | Nucleolar complex protein 3 homolog（NOC3L） | 2.035 | 0.002 |
| Succinate dehydrogenase iron-sulfur subunit, mitochondrial（SDHB） | 2.524 | 0.005 | HEAT repeat-containing protein 1（HEATR1） | 2.077 | 0.001 |
| Ribosome production factor 2 homolog（RPF2） | 2.515 | 0.003 | Sulfhydryl oxidase 2（QSOX2） | 2.065 | 0.008 |
| Protein disulfide-isomerase A5（PDIA5） | 2.001 | 0.042 | Delta(14)-sterol reductase LBR（LBR） | 2.511 | 0.001 |
| rRNA-processing protein FCF1 homolog（FCF1） | 2.082 | 0.012 | Calnexin（CANX） | 2.077 | 0.001 |
| Bifunctional 3-phosphoadenosine 5-phosphosulfate synthase 2（PAPSS2） | 2.081 | 0.002 | Membrane-associated progesterone receptor component 1（PGRMC1） | 2.077 | 0.016 |
| Mitochondrial import receptor subunit TOM70（TOMM70） | 2.078 | 0.006 | U3 small nucleolar ribonucleoprotein protein IMP4（IMP4） | 2.077 | 0.001 |
| Protein RRP5 homolog（PDCD11） | 2.011 | 0.007 | Protein lifeguard 3（TMBIM1） | 2.007 | 0.022 |
| Mitochondrial import inner membrane translocase subunit Tim8 A（TIMM8A） | 2.008 | 0.009 | Succinate dehydrogenase cytochrome b560 subunit, mitochondrial（SDHC） | 2.006 | 0.035 |
| GTP:AMP phosphotransferase AK3, mitochondrial（AK3） | 2.071 | 0.019 | Nucleolar complex protein 2 homolog（NOC2L） | 2.000 | 0.006 |

| **Table S2. Differentially expressed protein (down-regulated)** | | | | | |
| --- | --- | --- | --- | --- | --- |
| Protein Name | Passage40  /Passage0 | t test p value | Protein Name | Passage40  /Passage0 | t test p value |
| SLAIN motif-containing protein 2（SLAIN2） | 0.329 | 0.016 | PDZ and LIM domain protein 5（PDLIM5） | 0.497 | 0.007 |
| EF-hand domain-containing protein D1（EFHD1） | 0.499 | 0.014 | Mannose-1-phosphate guanyltransferase beta（GMPPB） | 0.495 | 0.000 |
| Cathepsin Z（CTSZ） | 0.499 | 0.022 | Tax1-binding protein 3（TAX1BP3） | 0.284 | 0.000 |
| RNA-binding protein FXR2（FXR2） | 0.499 | 0.000 | Peroxiredoxin-2（PRDX2） | 0.495 | 0.004 |
| Peptidyl-prolyl cis-trans isomerase NIMA-interacting 1（PIN1） | 0.498 | 0.001 | Tripartite motif-containing protein 3（TRIM3） | 0.495 | 0.008 |
| Histone H1.1（H1-1） | 0.499 | 0.015 | Plastin-1（PLS1） | 0.493 | 0.020 |
| Phosphoribosylformylglycinamidine synthase（PFAS） | 0.488 | 0.001 | MAP7 domain-containing protein 1（MAP7D1） | 0.491 | 0.009 |
| Profilin-3（PFN3） | 0.488 | 0.024 | Phosphomevalonate kinase（PMVK） | 0.488 | 0.014 |
| 5-3 exonuclease PLD3（PLD3） | 0.487 | 0.008 | Palladin（PALLD） | 0.491 | 0.005 |
| Angio-associated migratory cell protein（AAMP） | 0.491 | 0.002 | Translation machinery-associated protein 7（TMA7） | 0.497 | 0.028 |
| Protein phosphatase 3 catalytic subunit alpha（PPP3CA） | 0.487 | 0.001 | Platelet-activating factor acetylhydrolase IB subunit alpha2（PAFAH1B2） | 0.490 | 0.001 |
| Clustered mitochondria protein homolog（CLUH） | 0.483 | 0.014 | Mannose-1-phosphate guanyltransferase alpha（GMPPA） | 0.482 | 0.000 |
| Coiled-coil domain-containing protein 124（CCDC124） | 0.471 | 0.001 | Prefoldin subunit 5（PFDN5） | 0.481 | 0.003 |
| Tax1-binding protein 3（TAX1BP3） | 0.284 | 0.000 | Acylphosphatase-1（ACYP1） | 0.481 | 0.009 |
| Proteasome subunit beta type-6（PSMB6） | 0.483 | 0.004 | Cysteine protease ATG4B（ATG4B） | 0.487 | 0.028 |
| Probable E3 ubiquitin-protein ligase HERC1（HERC1） | 0.471 | 0.007 | Ubiquitin-like modifier-activating enzyme 1（UBA1） | 0.478 | 0.000 |
| MYG1 exonuclease（MYG1） | 0.471 | 0.003 | N-acetylglucosamine-6-sulfatase（GNS） | 0.463 | 0.002 |
| Ribonucleoside-diphosphate reductase large subunit（RRM1） | 0.329 | 0.002 | Immunity-related GTPase family Q protein（IRGQ） | 0.462 | 0.001 |
| Deoxyribonuclease-2-alpha（DNASE2） | 0.390 | 0.011 | Transcription factor p65（RELA） | 0.470 | 0.010 |
| Serine hydroxymethyltransferase, cytosolic（SHMT1） | 0.470 | 0.003 | Actin-related protein 2/3 complex subunit 1A（ARPC1A） | 0.461 | 0.000 |
| PRKC apoptosis WT1 regulator protein（PAWR） | 0.470 | 0.004 | Thioredoxin domain-containing protein 17（TXNDC17） | 0.480 | 0.001 |
| Huntingtin（HTT） | 0.463 | 0.000 | TOM1-like protein 2（TOM1L2） | 0.477 | 0.004 |
| Serine/threonine-protein phosphatase 2A 65 kDa regulatory subunit A beta isoform（PPP2R1B） | 0.449 | 0.006 | cAMP-dependent protein kinase catalytic subunit alpha（PRKACA） | 0.458 | 0.000 |
| Histidine--tRNA ligase, cytoplasmic（HARS1） | 0.459 | 0.001 | Ubiquitin-conjugating enzyme E2 T（UBE2T） | 0.458 | 0.001 |
| EH domain-containing protein 2（EHD2） | 0.442 | 0.000 | Ceramide transfer protein（CERT1） | 0.457 | 0.018 |
| 14-3-3 protein epsilon（YWHAE） | 0.460 | 0.000 | Tropomyosin alpha-4 chain（TPM4） | 0.456 | 0.000 |
| Transportin-2（TNPO2） | 0.431 | 0.005 | Sickle tail protein homolog（KIAA1217） | 0.456 | 0.014 |
| Cullin-3（CUL3） | 0.430 | 0.002 | Endophilin-A2（SH3GL1） | 0.444 | 0.000 |
| Rab5 GDP/GTP exchange factor（RABGEF1） | 0.428 | 0.005 | Pleckstrin homology-like domain family B member 1（PHLDB1） | 0.433 | 0.003 |
| Synembryn-A（RIC8A） | 0.426 | 0.000 | RNA demethylase ALKBH5 | 0.414 | 0.015 |
| AH receptor-interacting protein（AIP） | 0.423 | 0.001 | Major vault protein（MVP） | 0.412 | 0.001 |
| Centrosomal protein of 112 kDa（CEP112） | 0.421 | 0.001 | Caldesmon（CALD1） | 0.410 | 0.000 |
| RNA-binding protein 3（RBM3） | 0.421 | 0.005 | Calcineurin subunit B type 1（PPP3R1） | 0.410 | 0.007 |
| Transforming growth factor beta-1-induced transcript 1 protein（TGFB1I1） | 0.455 | 0.000 | Serine/threonine-protein phosphatase 4 regulatory subunit 1（PPP4R1） | 0.410 | 0.012 |
| Copper transport protein ATOX1（ATOX1） | 0.449 | 0.036 | Myeloid leukemia factor 2（MLF2） | 0.410 | 0.013 |
| Constitutive coactivator of PPAR-gamma-like protein 2（FAM120C） | 0.419 | 0.005 | SH3 domain-binding glutamic acid-rich-like protein 3（SH3BGRL3） | 0.398 | 0.002 |
| COP9 signalosome complex subunit 7b（COPS7B） | 0.418 | 0.003 | Rap1 GTPase-GDP dissociation stimulator 1（RAP1GDS1） | 0.240 | 0.001 |
| MARCKS-related protein（MARCKSL1） | 0.407 | 0.005 | Histone PARylation factor 1（HPF1） | 0.420 | 0.002 |
| Protein O-glucosyltransferase 2（POGLUT2） | 0.406 | 0.002 | Uncharacterized protein CXorf38（CXorf38） | 0.393 | 0.002 |
| L-lactate dehydrogenase A chain（LDHA） | 0.391 | 0.001 | SPARC | 0.325 | 0.007 |
| Kinesin-like protein KIF15（KIF15） | 0.385 | 0.015 | Myosin regulatory light polypeptide 9（MYL9） | 0.317 | 0.000 |
| Rho GTPase-activating protein 29（ARHGAP29） | 0.382 | 0.026 | EPM2A-interacting protein 1（EPM2AIP1） | 0.300 | 0.014 |
| Chromatin complexes subunit BAP18（BAP18） | 0.381 | 0.005 | Calcium/calmodulin-dependent protein kinase type 1（CAMK1） | 0.334 | 0.000 |
| Transcription factor BTF3（BTF3） | 0.379 | 0.023 | Prefoldin subunit 1（PFDN1） | 0.286 | 0.026 |
| SLC35A4 upstream open reading frame protein（SLC35A4） | 0.379 | 0.003 | Ubiquitin-conjugating enzyme E2 G1（UBE2G1） | 0.285 | 0.002 |
| Methylthioribose-1-phosphate isomerase（MRI1） | 0.285 | 0.000 | 40S ribosomal protein S4, Y isoform 1（RPS4Y1） | 0.265 | 0.000 |
| Tripeptidyl-peptidase 1（TPP1） | 0.402 | 0.047 | Profilin-1（PFN1） | 0.371 | 0.000 |
| Lysosomal protective protein（CTSA） | 0.369 | 0.000 | Acireductone dioxygenase（ADI1） | 0.265 | 0.000 |
| Triokinase/FMN cyclase（TKFC） | 0.353 | 0.001 | Histone H2A type 1-J（H2AC14） | 0.300 | 0.015 |
| Protein NDRG3（NDRG3） | 0.418 | 0.003 | Programmed cell death protein 4（PDCD4） | 0.257 | 0.002 |
| Vinexin（SORBS3） | 0.357 | 0.001 | Kinesin light chain 2（KLC2） | 0.357 | 0.000 |
| Putative peptidyl-tRNA hydrolase PTRHD1（PTRHD1） | 0.364 | 0.001 | Guanine nucleotide-binding protein subunit beta-4（GNB4） | 0.229 | 0.001 |
| Putative phospholipase B-like 2（PLBD2） | 0.348 | 0.011 | Calponin-2（CNN2） | 0.336 | 0.001 |
| Kinesin heavy chain isoform 5C（KIF5C） | 0.216 | 0.000 | Gamma-glutamyl hydrolase（GGH） | 0.188 | 0.001 |
| Parathymosin（PTMS） | 0.344 | 0.000 | Transgelin（TAGLN） | 0.068 | 0.000 |
| OCIA domain-containing protein 2（OCIAD2） | 0.219 | 0.004 | Aldehyde dehydrogenase family 16 member A1（ALDH16A1） | 0.271 | 0.002 |
| A-kinase anchor protein 12（AKAP12） | 0.344 | 0.000 | Epsin-3（EPN3） | 0.353 | 0.009 |
