# Supplementary material for: RNA m6A involves in regulation of oxidative stress and apoptosis may via NF-kB pathway in cadmium-induced lung cells
Source: Cell Death Discov. 2025 Jan 10;11:4. doi: 10.1038/s41420-024-02284-w (PMC11723944; doi:10.1038/s41420-024-02284-w)

**Immunoblot plot of representative m^6^A regulatory proteins in Passage 0, Passage 10, Passage 20, Passage 30 and Passage 40.**

**FYO**


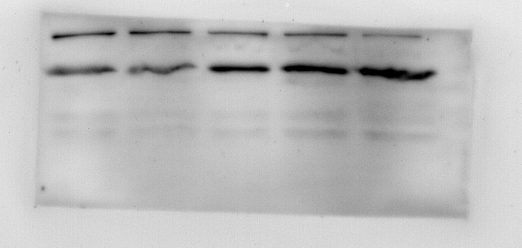


**ALKBH5**


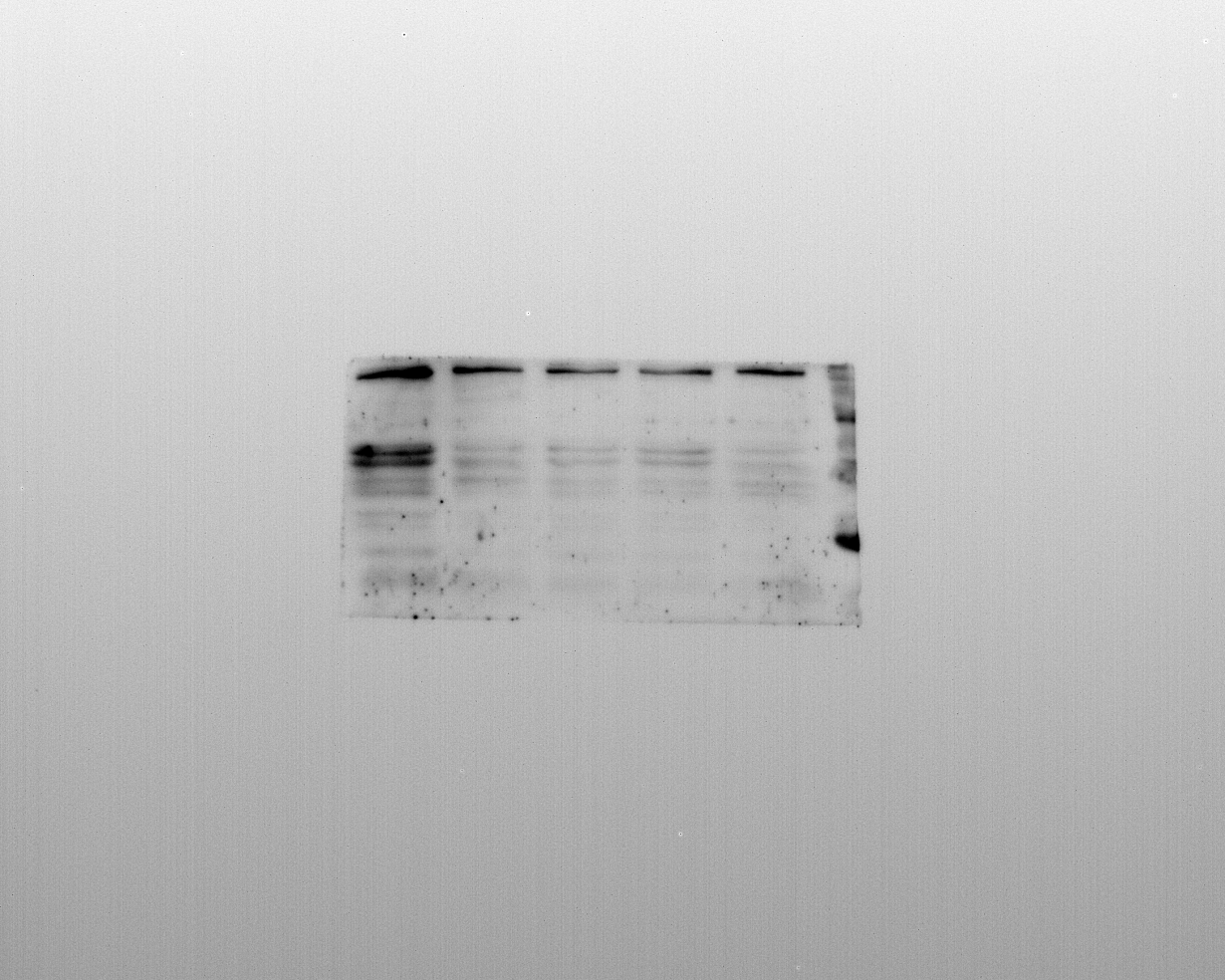


**YTHDC2**


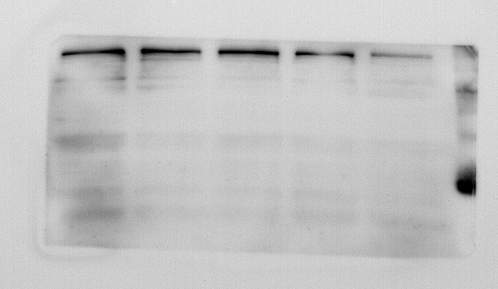


**GAPDH**


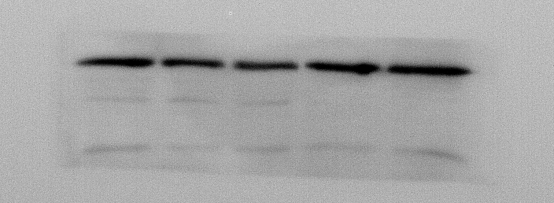


**Immunoblot plot of representative proteins in cells before and after DAA treatment in the nucleus.**

**NFKB p65**


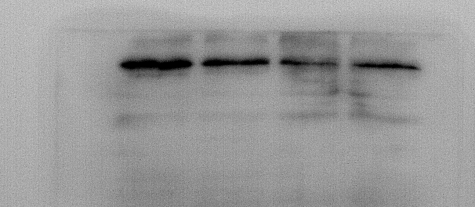


**NRF2**


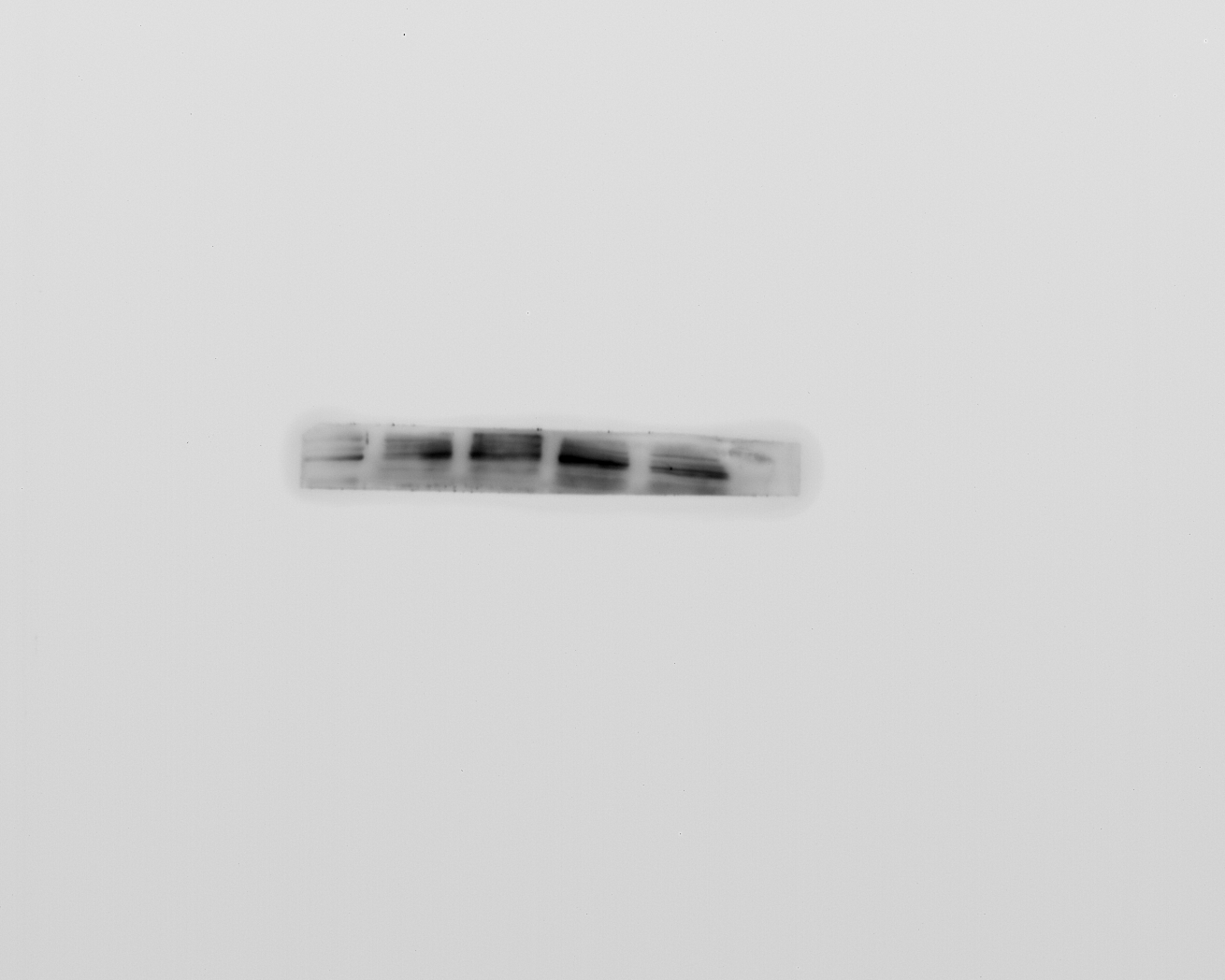


**GAPDH**


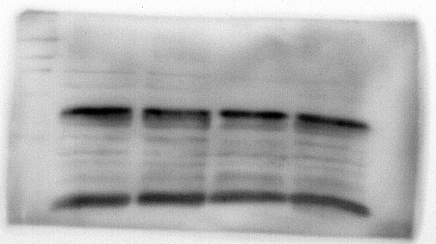


**Immunoblot plot of representative proteins in cells before and after DAA treatment in the cytoplasm.**

**NFKB p65**


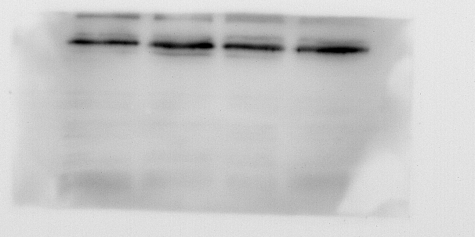


**NRF2**


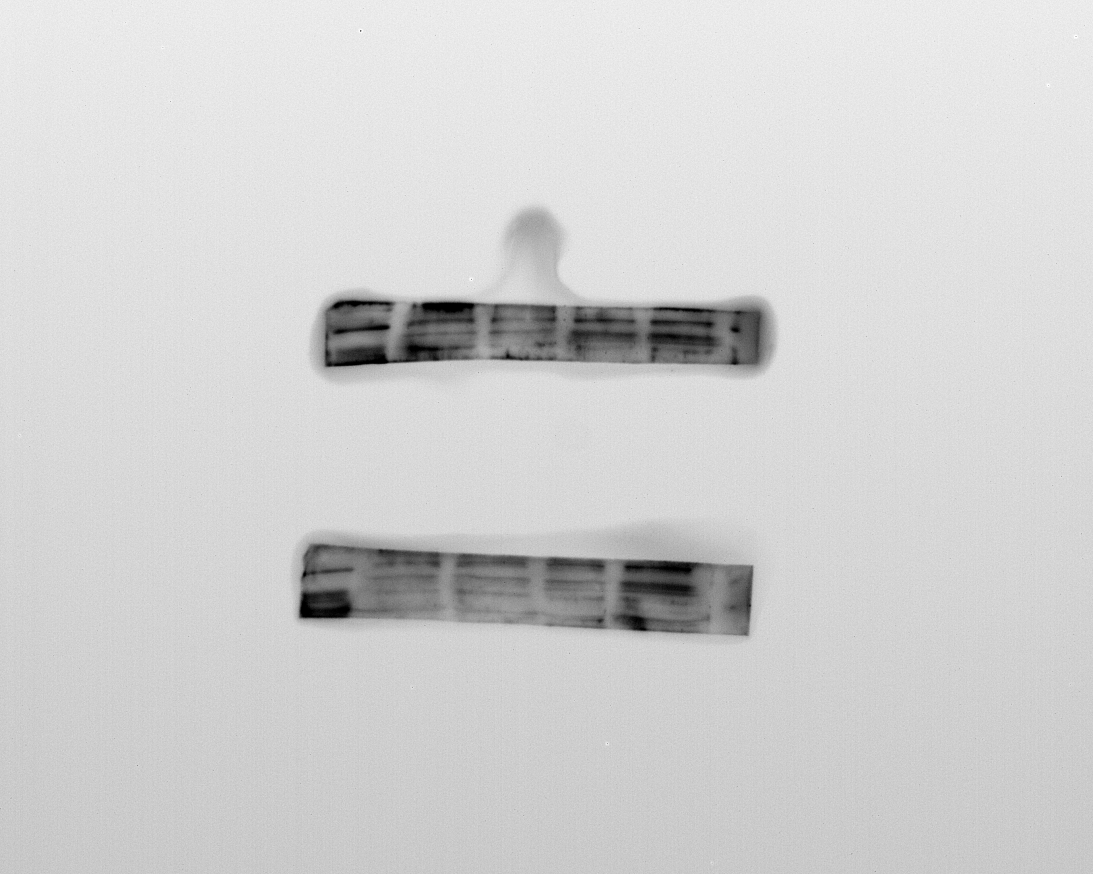


**GAPDH**


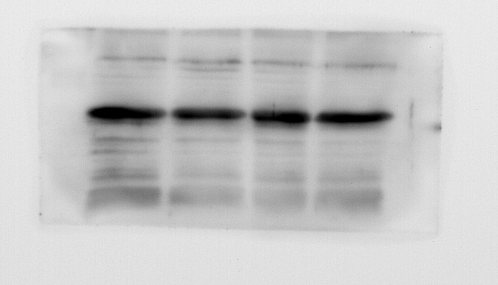


**Immunoblot plots of representative proteins associated with oxidative damage after DAA treatment.**

**SOD1**


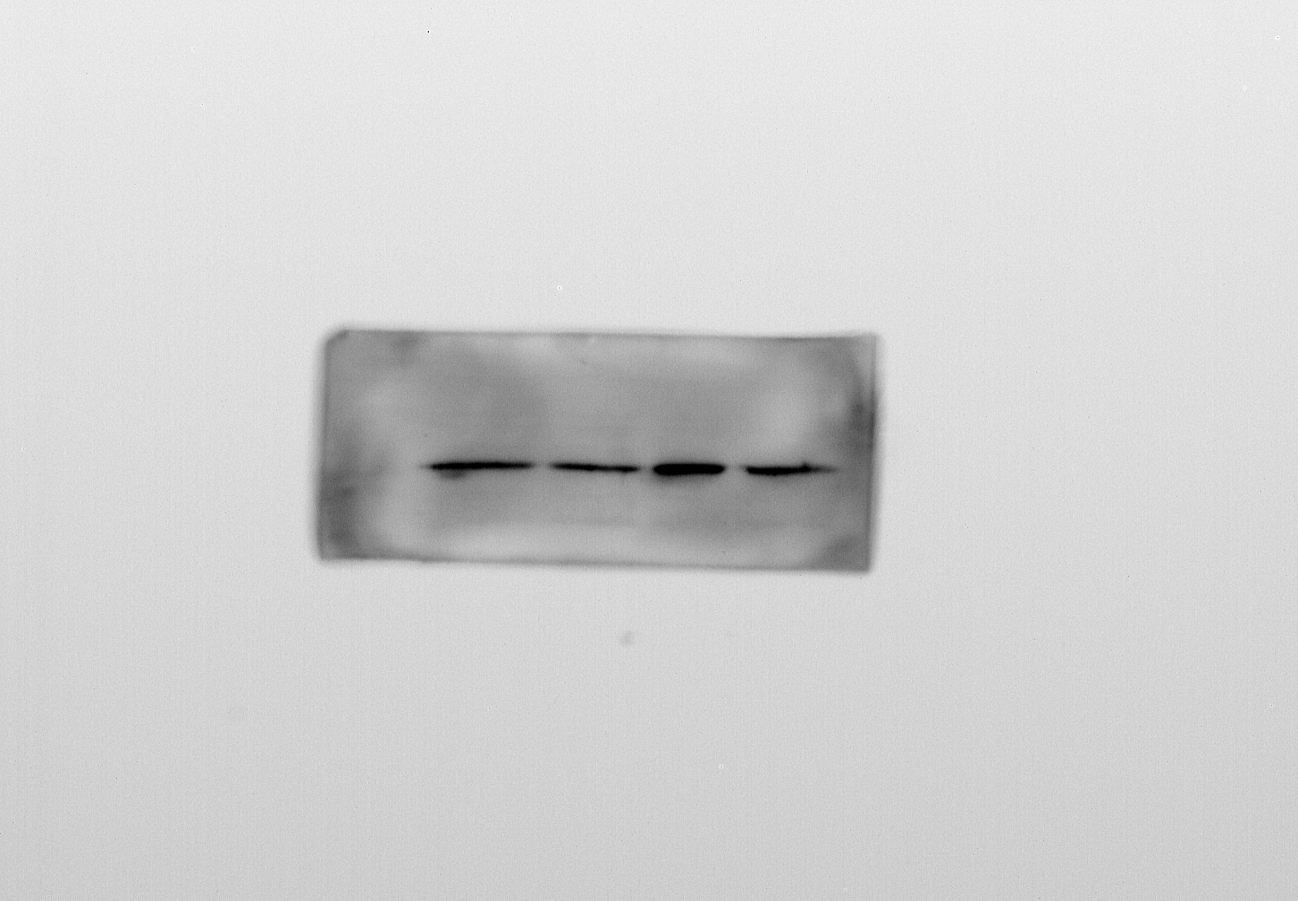


**NQO1**


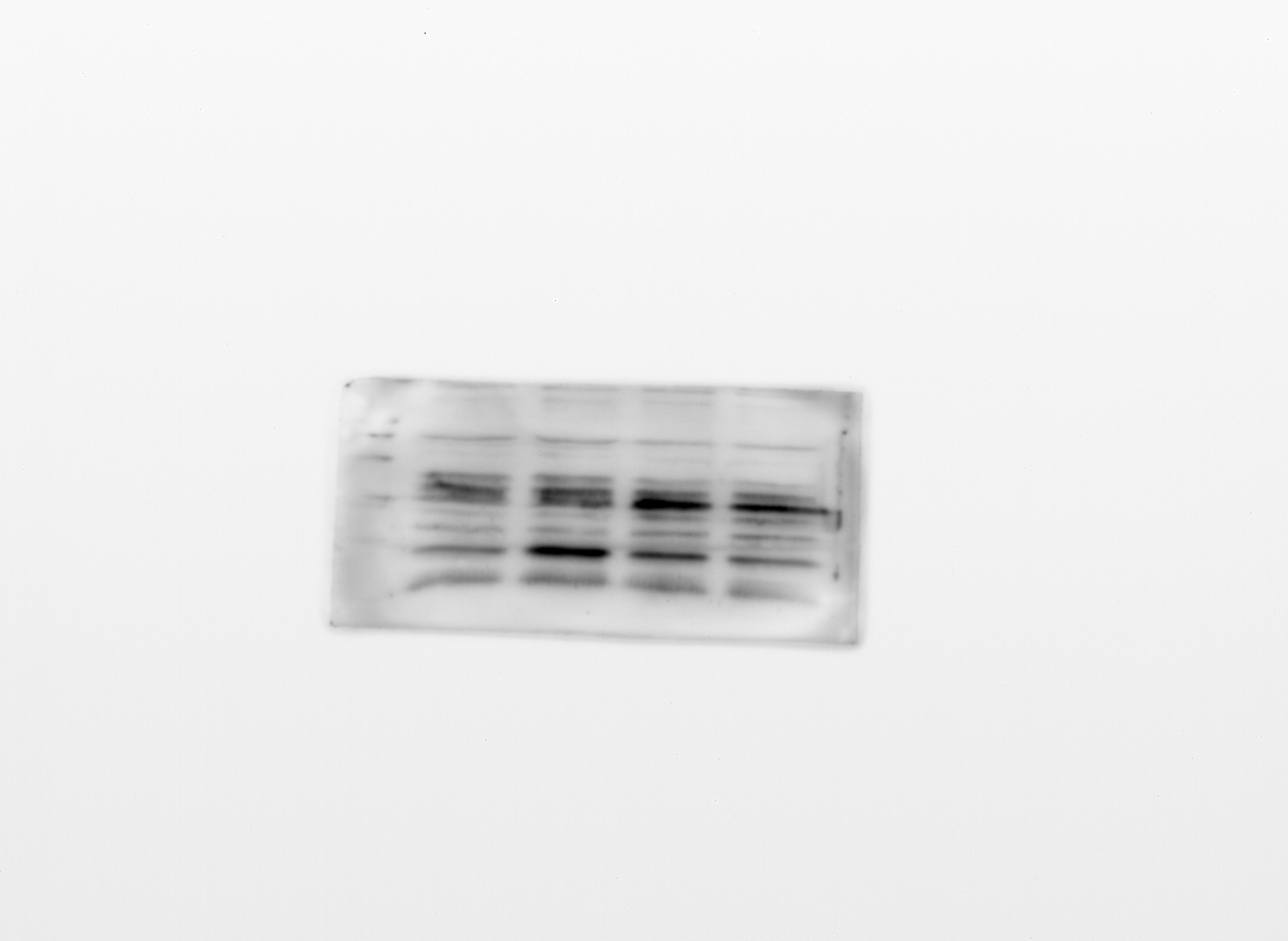


**BAX**


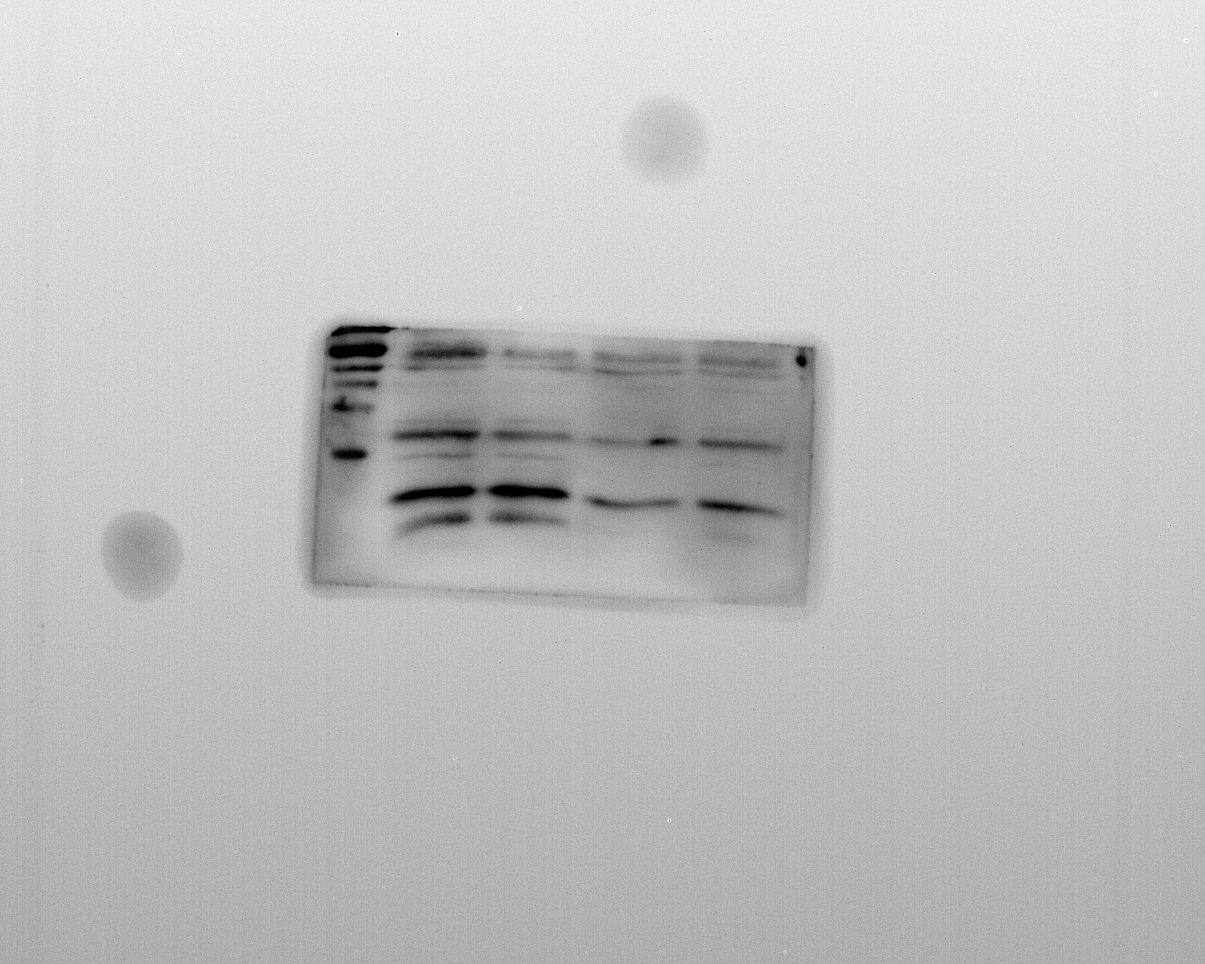


**GAPDH**


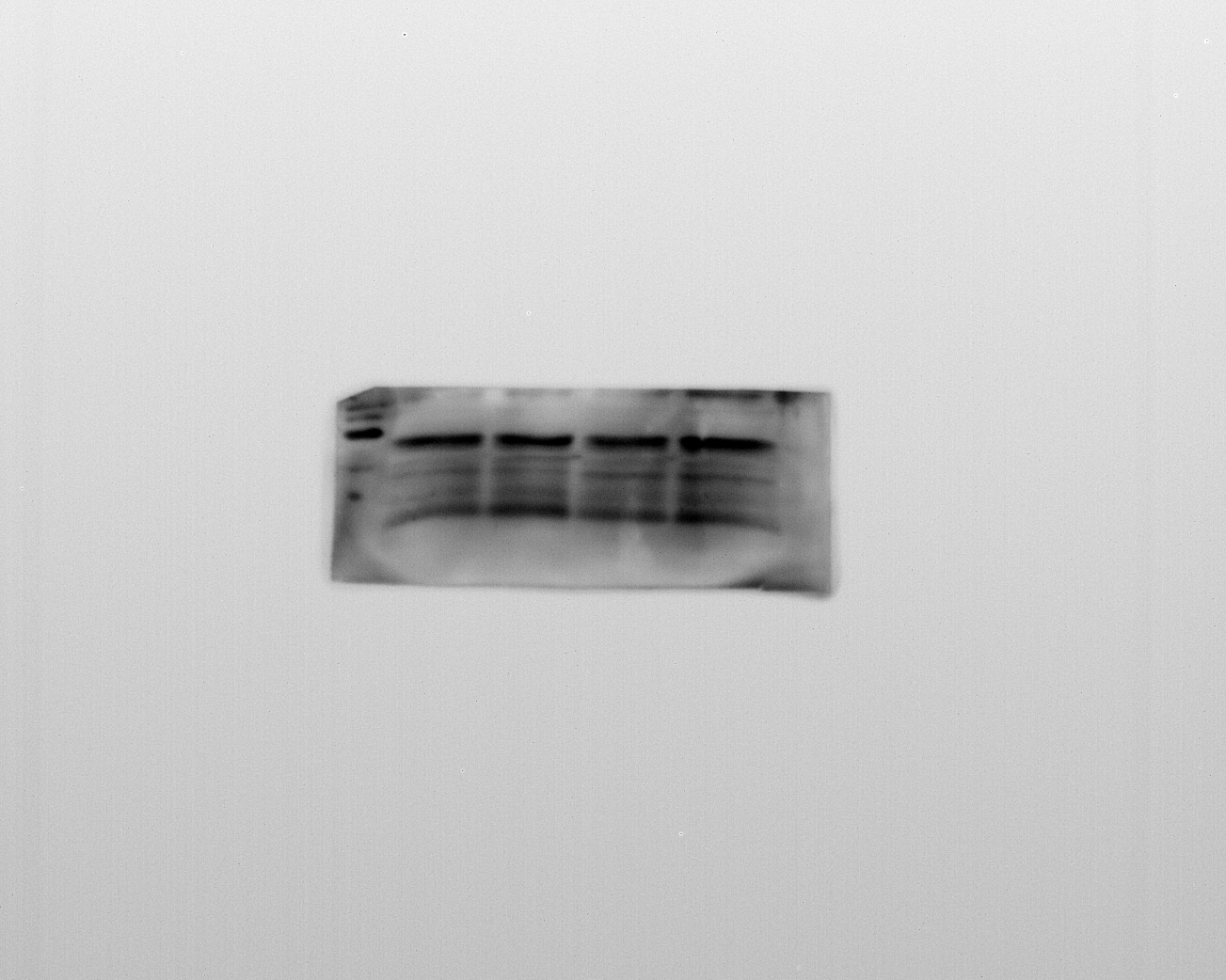


**Representative protein immunoblotting of proteins associated with oxidative damage in lung tissue after CdSO_4_ treatment.**

**GCLC**


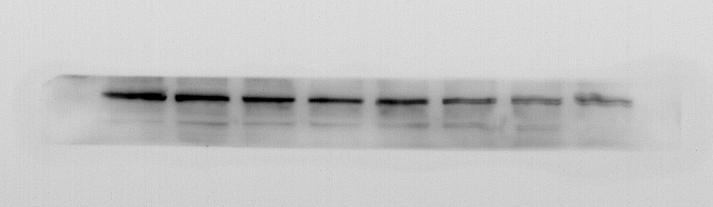


**NRF2**


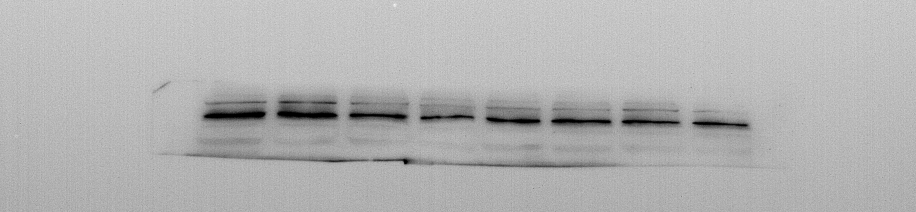


**NFKB p65**


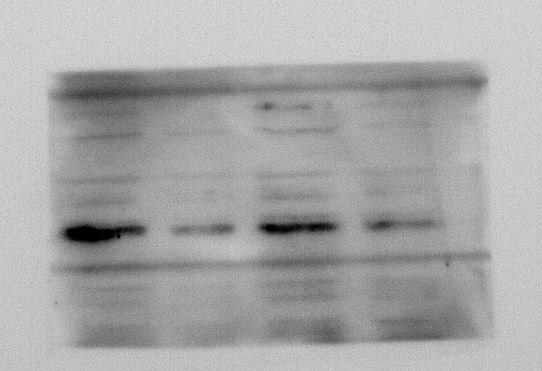


**HO-1**


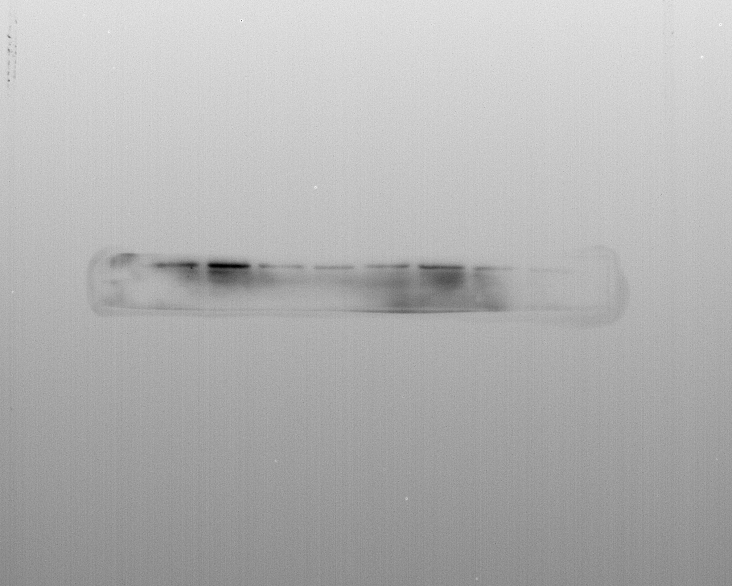


**NQO1**


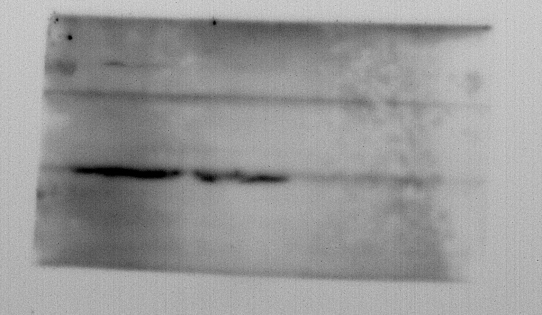


**BAX**


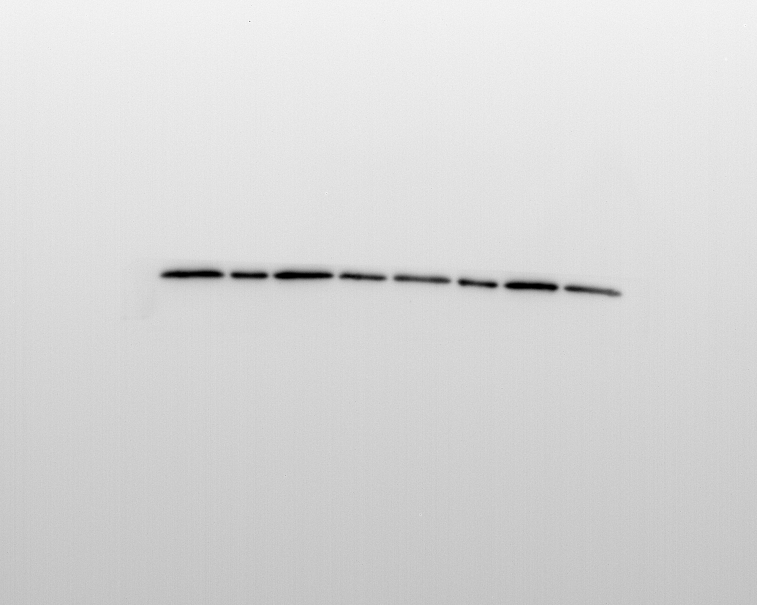


**SOD1**


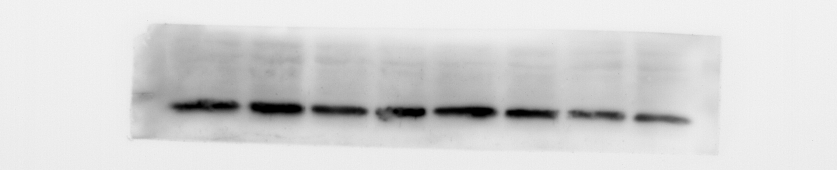


**SOD2**


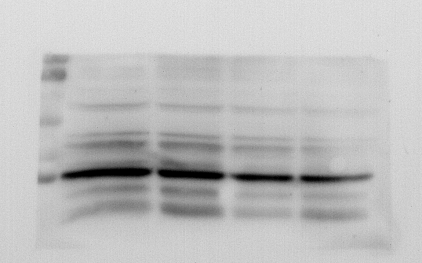


**GAPDH**


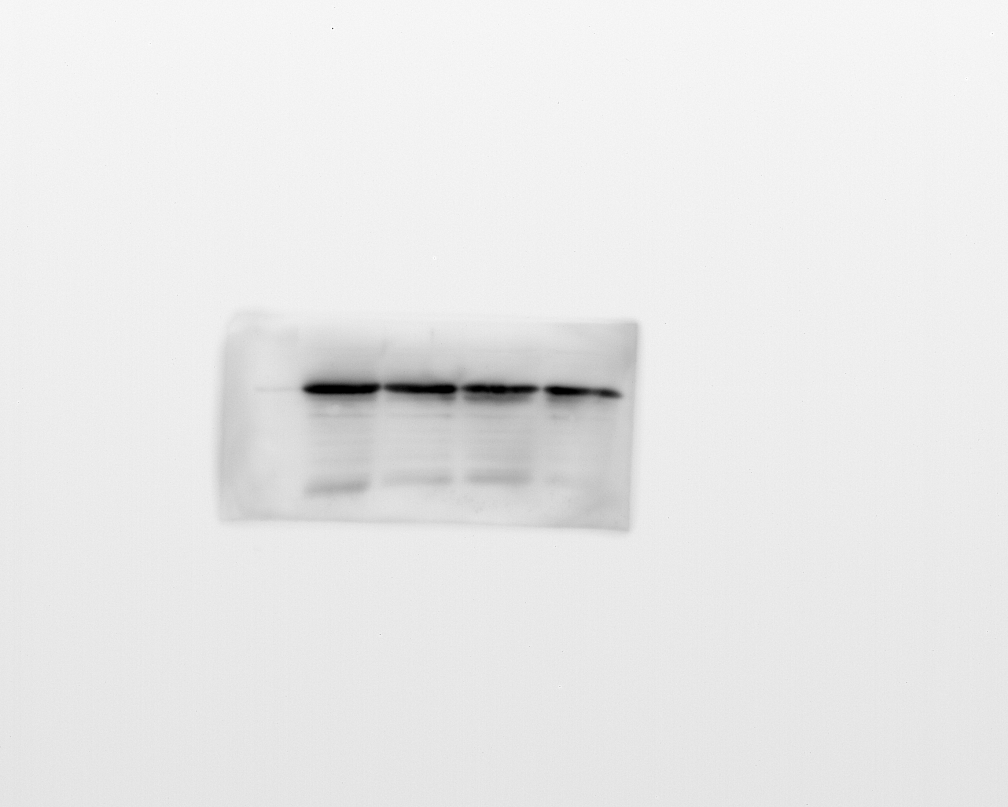


**Representative protein immunoblotting of m^6^A proteins in lung tissue after CdSO_4_ treatment.**

**FTO**


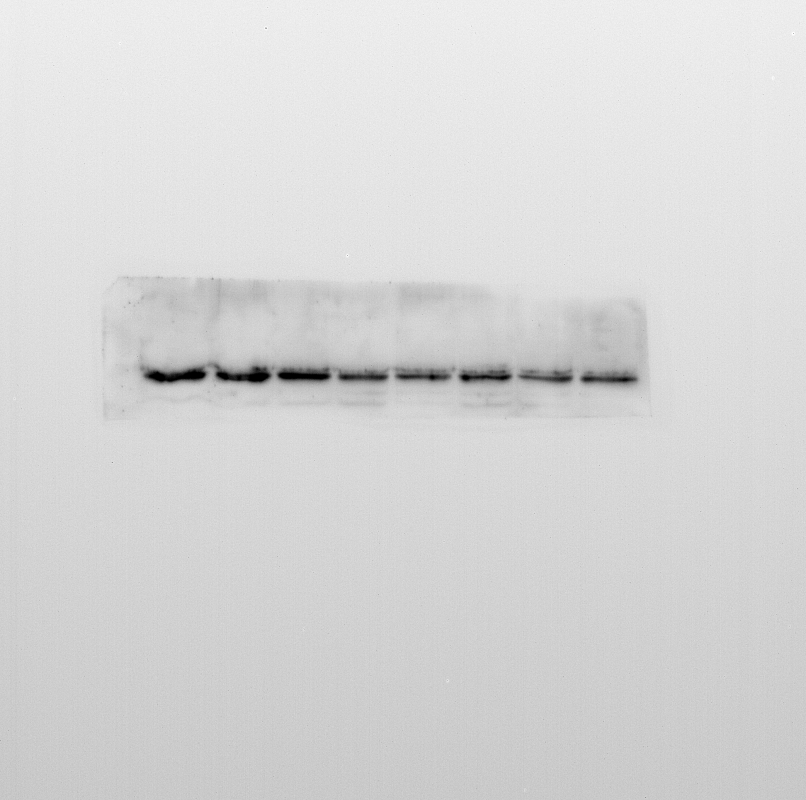


**ALKBH5**


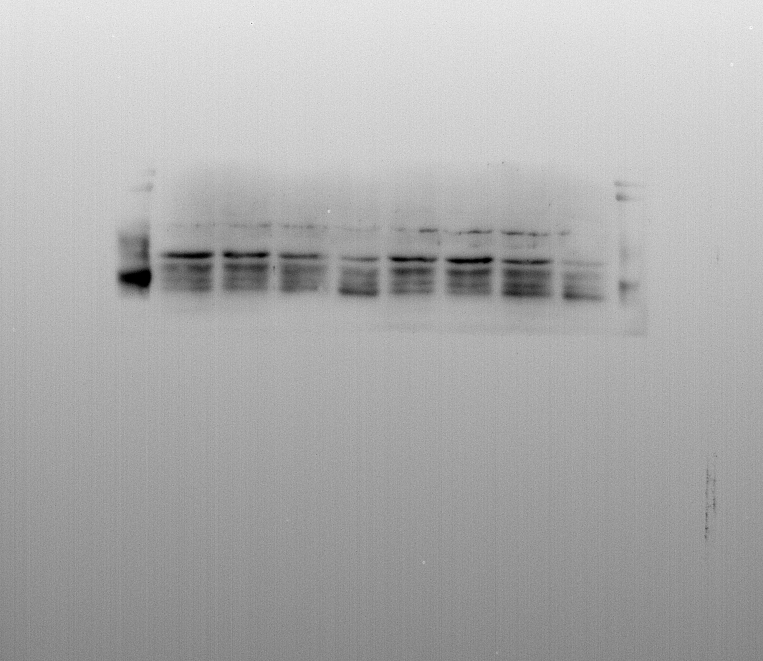


**YTHDC2**


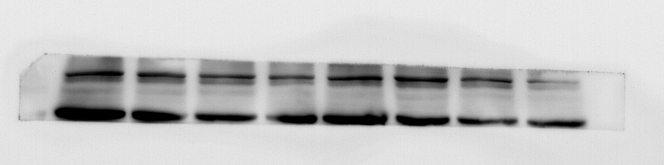


**GAPDH**


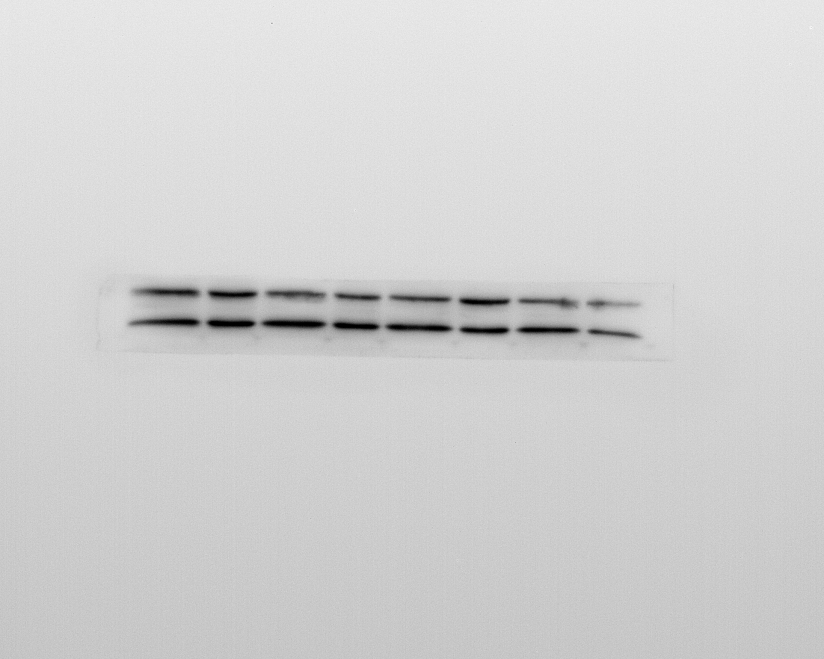

Supplement: Supplementary file 2 — Annex 2 [file 41420_2024_2284_MOESM2_ESM.docx]
